# Single-cell tracking of flavivirus RNA uncovers species-specific interactions with the immune system dictating disease outcome

**DOI:** 10.1038/ncomms14781

**Published:** 2017-03-14

**Authors:** Florian Douam, Gabriela Hrebikova, Yentli E. Soto Albrecht, Julie Sellau, Yael Sharon, Qiang Ding, Alexander Ploss

**Affiliations:** 1 Department of Molecular Biology, Princeton University, 110 Lewis Thomas Laboratory, Washington Road, Princeton, New Jersey 08544, USA

## Abstract

Positive-sense RNA viruses pose increasing health and economic concerns worldwide. Our limited understanding of how these viruses interact with their host and how these processes lead to virulence and disease seriously hampers the development of anti-viral strategies. Here, we demonstrate the tracking of (+) and (−) sense viral RNA at single-cell resolution within complex subsets of the human and murine immune system in different mouse models. Our results provide insights into how a prototypic flavivirus, yellow fever virus (YFV-17D), differentially interacts with murine and human hematopoietic cells in these mouse models and how these dynamics influence distinct outcomes of infection. We detect (−) YFV-17D RNA in specific secondary lymphoid compartments and cell subsets not previously recognized as permissive for YFV replication, and we highlight potential virus–host interaction events that could be pivotal in regulating flavivirus virulence and attenuation.

## Abstract (web summary)

Analysis of virus replication on a single-cell level is often hampered by a lack of specific or sensitive enough reagents. Here, Douam *et al*. use RNA-flow technique to track (+) and (−) strand RNA of yellow fever virus in hematopoietic cells in mouse models and identify virus-host interactions that affect disease outcome.

Infection by positive-sense RNA viruses, such as human immunodeficiency virus (HIV), hepatitis C virus (HCV) or flaviviruses such as dengue (DENV), Zika (ZIKV) and yellow fever viruses (YFV), remains a challenging global health issue^1^,^2^,^3^,^4^,^5^,^6^. For most of these pathogens, specific treatments or vaccines are unavailable. One major barrier to generating novel anti-viral strategies is our limited understanding of the nature, complexity and dynamics of interactions between these pathogens and the human host. In particular, it is incompletely understood how host–virus interactions regulate the molecular processes leading to virulence and disease or, conversely, immunogenicity.

Disease outcome is largely influenced by the dynamic interactions between a virus and the host immune system. Conventional experimental infection systems, specifically cell culture models, poorly reflect the complexity and heterogeneity of interactions that are also highly dependent on non-immune tissues. Although analysing immune responses in humans has provided important insights into virus–host biology, such clinical studies have multiple shortcomings. Usually only peripheral tissues, that is, blood, can be routinely accessed and perturbations, such as genetic alterations, are not possible. Furthermore, there is considerable intra- and inter-experimental variability due to heterogeneity of the study cohort and critical parameters like exposure time, dose and specific viral strain. *In vitro*, analysis is often limited to a few isolated human immune cell subsets as many lymphocytes require distinct conditions and cannot be maintained as a complex population in culture over sufficiently long periods to model the dynamics of an immune response. Thus, experimental animal models to delineate host–pathogen interactions remain critical. Genetically modified mice have been generated to evaluate the impact of multiple host factors on viral replication and pathogenesis^7^,^8^, and humanized mice, that is, mice engrafted with a human immune system (HIS)^9^, have emerged as a powerful platform to investigate viral interactions with the HIS (ref. 10).

Tracking viral antigen longitudinally in a complex, heterogeneous cell population can provide important insights into the interactions between a virus and the immune system, as well as how the spatio-temporal dynamics of such interactions might correlate with infection outcome. This is particularly relevant for studying pathogenesis and diseases that are, or are suspected to be, linked to active infection of HIS components by viruses such as Epstein-Barr virus^11^, HIV (ref. 12), DENV (ref. 13) or YFV (ref. 14).

Conventionally, fluorophore-labelled antibodies targeting viral proteins and immune cell proteins have been used to track viral antigens within the immune system. Using flow cytometry, a large number of single-cell events can be analysed at high-resolution in the context of heterogeneous cell populations. However, such an approach remains limited. Generating reliable and highly specific antibodies that target viral proteins similarly across multiple viral strains is a considerable challenge, especially in the context of emerging viruses. Moreover, even when reliable antibodies are available, low *in vivo* expression of the targeted viral proteins and lack of signal amplification result in poor signal sensitivity. Finally, targeting only viral proteins gives an incomplete picture as viral RNA molecules, independent of translation, can be involved in multiple interactions with components of the host immune system^15^. Hence, novel detection approaches, independent of viral proteins and applicable to multiple cell populations *in vivo*, are needed to more accurately characterize the spatio-temporal dynamics of virus interaction with cells of the immune system. The recent development of a novel flow cytometry application^16^, Prime RNA flow (RNA-flow), now allows the detection of RNA molecules by flow cytometry through strong signal amplification, opening new avenues for multiplex detection of RNA and proteins by flow cytometry. Here, we show that RNA-flow can be harnessed to track in a spatio-temporal manner positive (+) and negative (−) strand viral RNA within multiple cell subsets of the murine and human immune system.

A prototypic flavivirus, YFV is a positive-stranded RNA virus that causes a broad spectrum of symptoms in humans, ranging from subclinical manifestations to severe multi-organ dysfunction and death^17^. In contrast, the live-attenuated form of YFV, YFV-17D, which differs from its parental, highly pathogenic strain YFV-Asibi by only 32 amino acids^18^, represents one of the most efficient vaccines ever developed^19^,^20^. Although the molecular determinants regulating YFV pathogenesis or attenuation remain unknown, multiple lines of evidence suggest the immune system plays an important role^21^. Disease severity has been shown to correlate with high-levels of pro-inflammatory cytokines in fatal YFV cases^22^,^23^. The production and action of these cytokines, combined with the action of CD4+ and CD8+ lymphocytes, are thought to be responsible for the severe clinical manifestations observed during YFV infection^21^. YFV pathogenesis has also been shown to be both host and virus strain dependent^17^,^24^,^25^. Altogether, these observations suggest that differential interactions between YFV and the immune system influence the outcome of infection.

Discerning the spatio-temporal dynamics of YFV within the immune system will provide insights into how YFV interacts with the host immune system *in vivo*. In this study, we successfully applied RNA-flow technology to detect YFV-17D (+) and (−) strand RNA (YFV RNA-flow) within the murine and human immune system in different mouse models. We provide proof-of-concept for using this approach to analyse the spatio-temporal dynamics of viral RNA within complex immune cell populations during YFV-17D infection. Using these mouse models, we document distinct differences in the virus' ability to infect human and mouse hematopoietic cells with intact cell-intrinsic antiviral signalling capacity. Our data show that YFV-17D infection is antagonized by type I and III interferon (IFN) dependent antiviral defenses within the murine hematopoietic compartment, but the virus is able to overcome these innate defenses in human cells. Finally, we detect YFV-17D replication in specific immune compartments and cell subsets not previously described as supporting YFV-17D replication. Our analyses highlight particular virus–host interaction events as potential regulators of the mechanisms of YFV-17D pathogenesis.

## Results

### Detecting (+) and (−) sense YFV-17D RNA by vRNA flow

**Figure 1:**
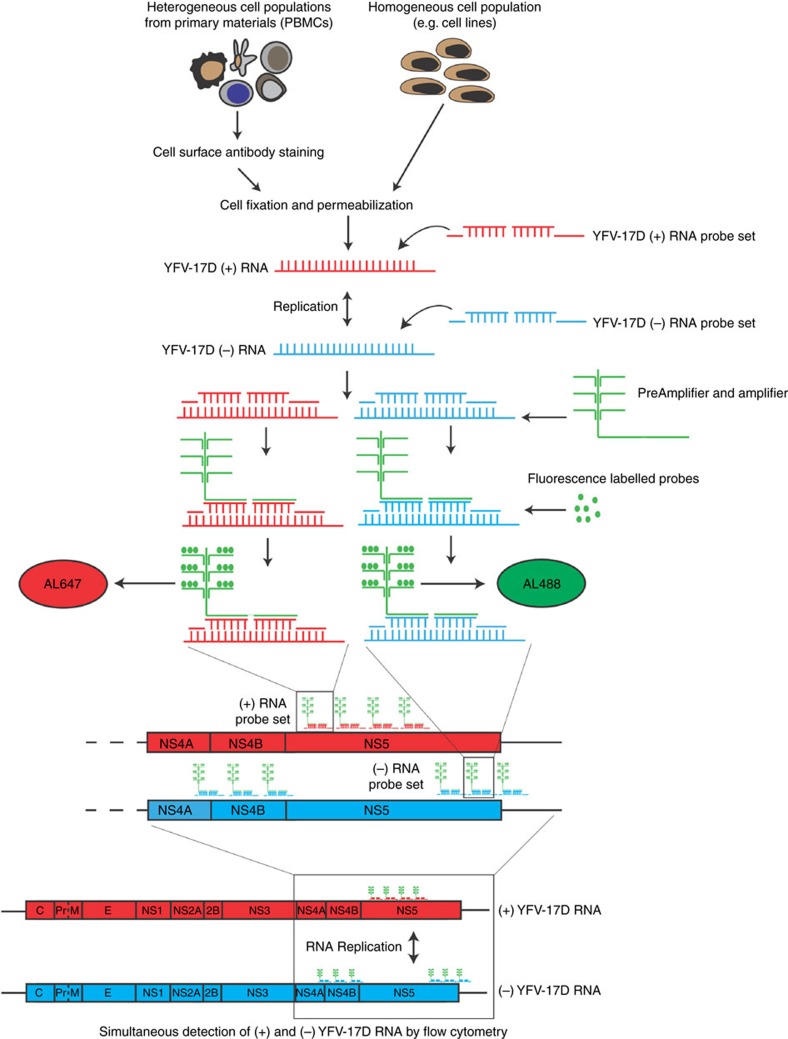
Harnessing flow cytometry application to profile viral RNA dynamics within complex cell populations. Schematic representation of the vRNA flow procedure. An Alexa 647-probe set was designed to target a 1753, bp sequence of the YFV-17D (+) RNA strand, located within the NS5 coding region. Additionally, an Alexa 488-probe set was designed to target a 929 and 840 bp sequence of the YFV-17D (−) strand, located, respectively, downstream and upstream of the 1,753 bp region targeted by the YFV-17D (+) RNA probe set on the (+) strand. Following hybridization of the probes to the (+) and (−) RNA, amplification of the fluorescence signal occurred via the use of PreAmplifier and Amplifier that bound the initial probes and served as binding sites for the fluorescent probes. Fluorescence signals of bound (+) and (−) YFV-17D RNA probe sets can then be detected by flow cytometry using the AL647/APC and AL488/FITC channels, respectively. Such a strategy thus allows simultaneous detection of both (+) and (−) strand YFV-17D RNA at a single-cell resolution within cell lines or within heterogeneous, primary cell populations.

To harness RNA-flow for the detection of YFV-17D (+) and (−) strand RNA (henceforth referred to as viral RNA flow or vRNA flow), we designed two sets of probes targeting a 1,700 bp region of either the (+) or (−) strand of YFV-17D RNA (henceforth referred to as (+) and (−) probe sets). Following cell permeabilization and probe hybridization, pre-amplifier and amplifier bind, respectively, the target probes and the pre-amplifier, generating multiple binding sites for fluorescent-labelled probes on a single target probe (Fig. 1). This amplification step enables sensitive detection of target probe hybridization to viral RNA. Since the (+) and (−) probes are conjugated to different fluorophores (Alexa (AL) 647 and AL488, respectively), (+) and (−) viral RNA, indicative of viral replication, can be simultaneously detected at the single-cell level.

**Figure 2:**
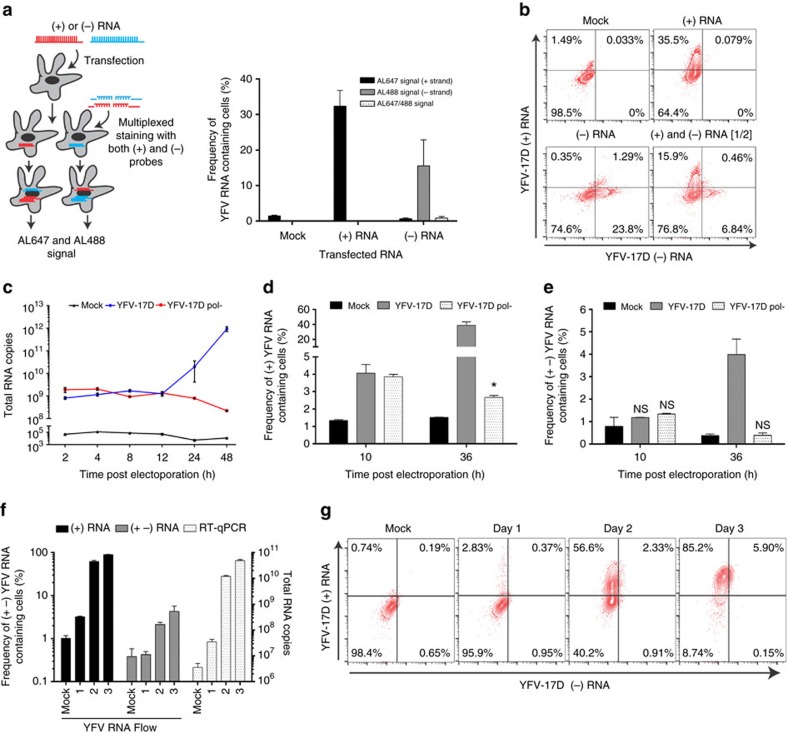
YFV-17D (+) and (−) RNA can be specifically and sensitively detected by distinct probe sets. (**a**) Assessing the specificity of the YFV-17D RNA probe sets. HEK293T cells were transfected with a small [NS4A-3′UTR] YFV-17D RNA of (+) or (−) sense. Six hours post-transfection, cells containing either (+) or (−) RNA were processed using the vRNA flow procedure and stained with both the (+) and (−) probe sets. For each transfection (mock, (+) RNA and (−) RNA), a fraction of YFV-17D RNA-containing cells emitting an AL647 signal (bound (+) probe set, black), an AL488 signal (bound (−) probe set, grey) or both (dotted white) was quantified (mean±s.d.; *n*=3). (**b**) Representative FACS plots of HEK293T cells transfected with a [NS4A-3′UTR] YFV-17D RNA of (+) or (−) sense and processed using the vRNA flow. Cells transfected with (+) were mixed in equal ratios with cells transfected with (−) YFV-17D RNA and processed using the same procedure (lower right panel). (**c**) Quantification of YFV-17D and YFV-17D pol- positive-sense intracellular RNA over time by RT-qPCR following RNA electroporation of Huh7.5 (mean±s.d.; *n*=3). (**d**,**e**) Quantification of Huh7.5 containing positive (+) (**d**) or positive and negative (+−) (**e**) RNA of the parental YFV-17D (grey) and YFV-17D pol- (white dotted) at 10 and 36 h post electroporation using vRNA flow (mean±s.d.; *n*=3; *P<0.05, NS non-significant; Two-way ANOVA test). Representative FACS plots can be seen in Supplementary Fig. 1c. (**f**) Quantification of Huh7.5 containing positive (+, black) or positive+negative (+−, grey) RNA at days 1,2,3 post YFV-17D infection. Replication kinetics were compared to detection of YFV-17D positive RNA by RT-qPCR (white dotted). (mean±s.d.; *n*=3). (**g**) Representative FACS plots of infected Huh7.5 at different times post infection and processed using vRNA flow.

To assess the specificity of our (+) and (−) probe sets, we transfected HEK293T cells with *in vitro* transcribed RNA fragments derived from (+) or (−) YFV-17D RNA coding for the [NS4A-3′UTR] sequence. Six hours post-transfection, cells were processed following the vRNA flow procedure and incubated with both (+) and (−) probe sets. The probe sets were highly specific for their respective targets with no noticeable cross-reactivity (Fig. 2a,b). To further ascertain the specificity of the assay, we generated a replication-deficient YFV-17D strain (YFV-17D pol−) by mutating the residues 3172 and 3173 (GDD to GSA) in the catalytic site of the RNA-dependent RNA polymerase (RdRP) as previously described^26^. This mutation rendered YFV-17D unable to replicate and propagate *in vitro* as evidenced by RT-qPCR (Fig. 2c and Supplementary Fig. 1a) and the absence of a cytopathic effect (Supplementary Fig. 1b) following parallel electroporation of human hepatoma Huh7.5 cells with either YFV-17D or YFV-17D pol− RNA. Similarly, we assessed our (+) and (−) strand probe sets following electroporation of *in vitro* transcribed RNA of these two genomes into Huh7.5 cells. In cells transfected with the replication incompetent YFV-17D genome, only (+) RNA was detected at 10 h and, to a lesser extent, 36 h post electroporation (Fig. 2d,e and Supplementary Fig. 1c). In contrast, cells transfected with the unmodified YFV-17D genome, which produces a (−) strand intermediate to generate more viral genomes, both RNA species were detected 36 h post electroporation (Fig. 2d,e), confirming the specificity of our probe sets. Finally, we applied vRNA flow to assess the dynamics of (+) and (−) viral RNA in an infection context. In Huh7.5 cells infected with YFV-17D, we observed an increasing frequency of Huh7.5 cells bearing (+) alone, or both (+) and (−) strand YFV-17D RNA over three days. The frequency of cells carrying (+) viral RNA scaled with the increasing level of intracellular YFV-17D RNA across the whole population of cells as detected by RT-qPCR (Fig. 2f,g).

### IFN signalling in immune cells controls YFV-17D in mice

**Figure 3:**
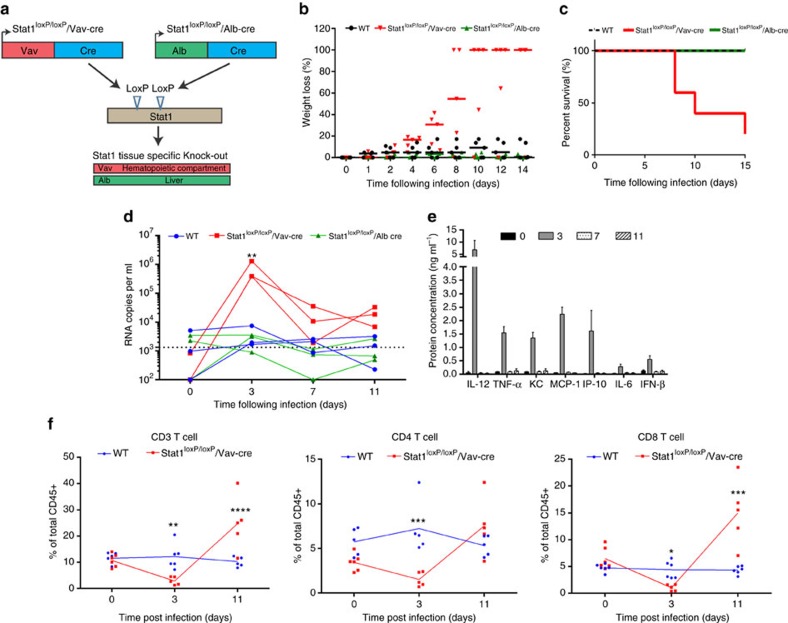
Stat1-specific knock-out in the murine hematopoietic compartment induces severe pathogenesis and death following YFV-17D infection. (**a**) Schematic representation of the two tissue-specific Cre-mediated Stat1 knock-out systems generated in C57BL/6 mice and used in this study. (**b**) Weight loss of C57BL/6 mice (WT), stat1^loxP/loxP^/Alb-cre and stat1^loxP/loxP^/Vav-cre mice over the course of YFV-17D infection. Medians are shown. 100% weight loss symbolizes death of the respective animal (*n*=5 per group). (**c**) Survival of C57BL/6 mice (WT), stat1^loxP/loxP^/Alb-cre and stat1^loxP/loxP^/Vav-cre mice following YFV-17D infection (*n*=5 per group). (**d**) Serum viremia of C57BL/6 mice (WT), stat1^loxP/loxP^/Alb-cre and stat1^loxP/loxP^/Vav-cre mice over time following YFV-17D infection. Positive-strand RNA copies per ml were quantified by RT-qPCR. Limit of detection (dotted line) is shown (*n*=3 per group; **P<0.01, ns non-significant; Two-way ANOVA test). (**e**) Protein concentration of a panel of pro-inflammatory cytokines in the serum of stat1^loxP/loxP^/Vav-cre mice at days 0, 3, 7 and 11 post infection. Only the cytokines displaying a significant concentration change over time are shown. The complete panel of tested cytokines is shown in Supplementary Fig. 3a (mean±s.d.; *n*=3). (**f**) Frequency of peripheral CD3+ T cells (left), CD3+ CD4+ T cells (middle) and CD3+ CD8+ T cells (right) over time following infection of WT (blue) and stat1^loxP/loxP^/Vav-cre mice (red) with YFV-17D. Frequencies are expressed as percentage of total murine CD45+ cells (*n*=5 per group). Lines are linking the mean of each time point (*n*=5 per group; *P<0.05, **P<0.01, ***P<0.001, ****P<0.0001; Two-way ANOVA test).

In mice, YFV-17D infection is strongly attenuated and rapidly cleared by the IFN response^25^,^27^,^28^. Indeed, whole-body knockout of the type I and II IFN receptors renders mice highly susceptible to infection with YFV-17D (ref. 27). A considerable caveat of this knock-out strain is the strong impairment of both adaptive immunity and cell-intrinsic innate immunity across all tissues. On the basis of evidence that interactions between YFV and immune system components dictate disease outcome and the fact that the murine IFN response is critical for controlling YFV-17D infection, we hypothesized that blunting IFN signalling in immune cell compartments would suffice to render mice susceptible to viral infection. To create mice with impairment of IFN signalling specifically in hematopoietic cells, we intercrossed mice harbouring a floxed Stat1 allele (stat1^loxP/loxP^) with mice expressing a Cre recombinase under the control of a Vav promoter (Fig. 3a). The resultant stat1^loxP/loxP^/Vav-cre mice were highly susceptible to YFV-17D infection, indicated by rapid weight loss (Fig. 3b), progressively worsening clinical appearance (Supplementary Fig. 2a) and death within two weeks of infection (Fig. 3c). In contrast, wild-type mice (WT) and mice harbouring a stat1-specific knock-out in hepatocytes (stat1^loxP/loxP^/Alb-cre in which Cre recombinase is expressed under the control of an albumin promoter), did not show any overt clinical phenotype or mortality. These results demonstrate that depletion of IFN signalling in the hematopoietic compartment recapitulates the clinical features observed in germline type I/II IFN knock-out mice, thus highlighting the hematopoietic compartment as a critical regulator of YFV infection *in vivo*. Consistent with the observed clinical phenotype, stat1^loxP/loxP^/Vav-cre, but not WT and stat1^loxP/loxP^/Alb-cre mice, displayed a sharp increase in YFV-17D RNA in the serum 3 days post infection followed by a decline in viremia between days 3 and 7 post infection (Fig. 3d). This viremia profile was confirmed in independent cohorts (Supplementary Fig. 2b). Elevated serum viremia at day 3 post infection correlated with a strong increase in the serum concentration of pro-inflammatory cytokines, including TNF-α, MCP-1, IP-10, IL-6 and KC (Fig. 3e and Supplementary Fig. 2c). At the same time, we observed a decrease in the frequencies of both peripheral CD3+ CD4+ and CD8+ T cells (Fig. 3f) in stat1^loxP/loxP^/Vav-cre but not control animals. Only the CD8+, but not CD4+, T cell pool expanded by day 11 to comprise ∼15% of all CD45+ lymphocytes. Frequencies of other major lymphocyte populations, specifically natural killer (NK) cells and B cells, exhibited distinct patterns in the peripheral blood during the course of infection in stat1^loxP/loxP^/Vav-cre versus stat sufficient mice (Supplementary Fig. 2d,e).

**Figure 4:**
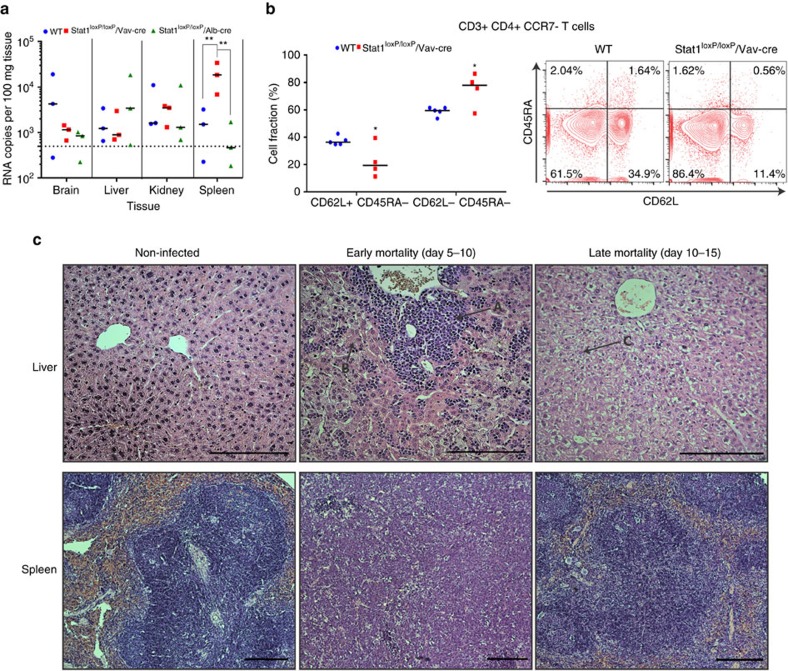
Stat1-specific knock-out in the murine hematopoietic compartment enables liver damage and extensive replication in the spleen. (**a**) Viral loads within multiple tissues of C57BL/6 mice (WT), stat1^loxP/loxP^/Alb-cre and stat1^loxP/loxP^/Vav-cre mice at time of killing (day 11) following YFV-17D infection. Positive-strand RNA copies per 100 mg of tissues were quantified by RT-qPCR. Medians and limit of detection (dotted line) are shown (*n*=3 per group; **P<0.01; Two-way ANOVA test). (**b**) Level of CD45RA and CD62L expression among splenocyte-residents CD3+ CD4+ CCR7− at the time of killing (day 11 post infection) following infection of WT (blue) and stat1^loxP/loxP^/Vav-cre mice (red) with YFV-17D. Frequencies of CD62+ CD45RA− and CD62L− CD45RA− populations are displayed in the left panel. Medians are shown. A representative FACS plot is shown in the right panel (*n*=4–5 per group; *P<0.05; Two-way ANOVA test). (**c**) Hematoxylin and Eosin (H&E) staining of mouse liver (× 20 magnification) and spleen (× 10 magnification) tissue sections from non-infected and YFV-17D infected stat1^loxP/loxP^/Vav-cre mice experiencing early (days 3–5 post infection) or late death (days 10–15 post infection). For each experimental condition (non-infected or infected) and tissue (Liver and Spleen), six tissue sections from three biological replicates (three animals) were examined. Histopathological manifestations observed in infected animal tissues were absent from all examined non-infected animals, and were representative of three biological replicate, for a given type of tissue. Scale bar (200 μm) is indicated for each picture. A, marked inflammation; B, piecemeal necrosis; C, hydropic changes.

We next analysed YFV-17D RNA across different tissues. YFV-17D viral load was similar in the brain, liver and kidney of WT mice and mice with targeted disruptions of stat in either the hematopoietic compartment or the liver. In contrast, viral RNA was significantly more abundant in the spleen of stat1^loxP/loxP^/Vav-cre mice, suggesting that this secondary lymphoid organ is the main site for YFV-17D replication in the absence of intact antiviral signalling (Fig. 4a). Consistently, more CD3+ CD4+ T cells, but not CD8+ T cells, were found to display an effector memory phenotype (CD3+CD4+CCR7−CD45RA−CD62L−) at day 11 post infection in the spleen of stat1^loxP/loxP^/Vav-cre mice (Fig. 4b and Supplementary Fig. 3a).

Histological analysis showed that stat1^loxP/loxP^/Vav-cre mice experiencing early mortality (days 5–10 post infection) had marked liver damage and severe inflammation, associated with extensive lymphocyte infiltration and piecemeal necrosis (Fig. 4c and Supplementary Fig. 3b,c). The most severe course of YFV-17D infection in stat1^loxP/loxP^/Vav-cre mice was also associated with a severe disruption of splenic organization. Stat1^loxP/loxP^/Vav-cre mice experiencing late mortality (days 10–15 post infection) displayed liver tissue with disarray and hydropic changes and moderate loss of splenic architecture (Fig. 4c and Supplementary Fig. 3b,c). These severe histopathologic phenotypes were not observed in clinically asymptomatic stat1 sufficient mice injected with YFV-17D. Together, these observations support the idea that specific interactions between YFV-17D and hematopoietic components, facilitated by loss of IFN signalling in immune cells, result in virally induced immunopathology. Hence, stat1^loxP/loxP^/Vav-cre mice are a useful model for assessing how viral interaction dynamics are impacted by the ability of hematopoietic cells to initiate a primary response to infection and how these dynamics correlate with pathogenesis in mice.

### Hematopoietic stat1 deficiency augments YFV-17D replication

**Figure 5:**
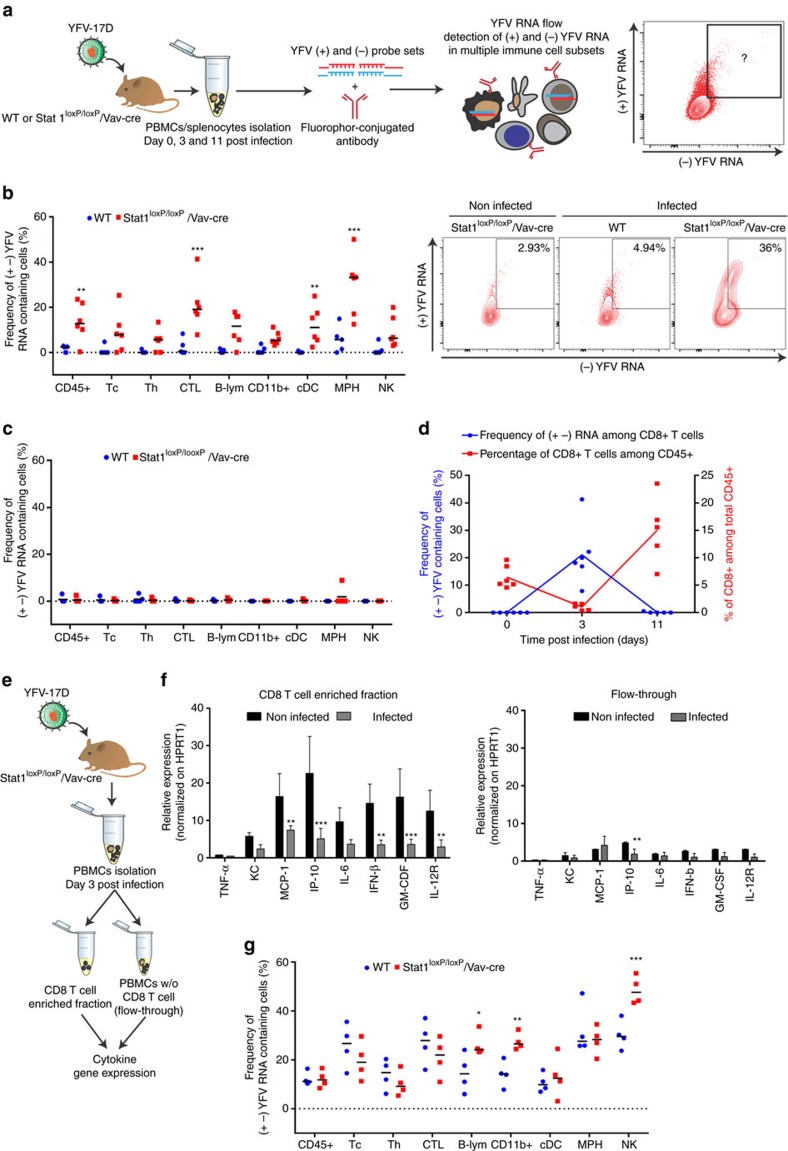
Stat1 deletion in the hematopoietic compartment impacts the viral replication dynamics of YFV-17D in the spleen of animals. (**a**) Schematic representation of the experimental procedure for characterizing the spatio-temporal profiles of YFV-17D replication (cells containing both the positive and negative YFV-17D RNA) in the murine immune system using vRNA flow. (**b**,**c**) Frequency of cells containing both (+) and (−) YFV-17D RNA in multiple subsets of peripheral murine CD45+ cells of WT (blue) and stat1^loxP/loxP^/Vav-cre mice (red) at day 3 (**b**) and 11 (**c**) post infection (*n*=5–6 per group; **P<0.01, ***P<0.001; Two-way ANOVA test). Representative FACS plots displaying presence of YFV-17D replication intermediates in peripheral CD3+ CD8+ T cell populations of WT and stat1^loxP/loxP^/Vav-cre mice at day 3 post infection is shown at the right of **b**. (**d**) Comparison of the evolution of CD8+ T cell frequency in the peripheral blood of stat1^loxP/loxP^/Vav-cre mice (red) with the frequency of peripheral CD8+ T cells containing (+) and (−) YFV-17D RNA (blue) over the course of infection (*n*=5–6 per group). (**e**) Schematic representation of the procedure of peripheral CD8+ T cell enrichment from the blood of stat1^loxP/loxP^/Vav-cre mice. (**f**) Relative gene expression of selected cytokines and cytokine receptors within peripheral CD3+CD8+ T cells (CD8+ enriched fraction) and PBMCs depleted for CD3+CD8+ T cells (flow-through) isolated from non-infected and infected stat1^loxP/loxP^/Vav-cre mice (day 3 post infection, *n*=3). Gene expression was normalized to the expression level of murine HPRT1 (mean±s.d.; *n*=5, **P<0.01, ***P<0.001; Two-way ANOVA test). (**g**) Frequency of cells containing both (+) and (−) YFV-17D RNA in multiple subsets of spleen-resident murine CD45+ cells of WT (blue) and stat1^loxP/loxP^/Vav-cre mice (red) at day 11 post infection (*n*=5–6 per group; **P<0.01, ***P<0.001; Two-way ANOVA test). Frequencies of YFV RNA-bearing cells were normalized to background staining in the equivalent cell populations in spleens of non-infected animals. Tc, CD3+ T cells; Th, CD4+ T cells; CTL, CD8+ T cells; B-lym, CD19+ B cells; CD11b+, CD3− CD19− CD11b+ cells; cDC, CD3− CD19− CD11c+ conventional dendritic cells; MPH, CD3− CD19− CD11b+ F4/80+ macrophages; NK, CD3− CD19− CD161+ NK cells. Details in population gating are described in materials and methods.

We then employed vRNA flow to assess whether the severe pathogenesis induced by stat1 knock-out in murine hematopoietic cells correlated with a differential interaction between YFV-17D and the murine immune system. Combining the use of our (+) and (−) probe sets with fluorophore-conjugated antibodies, we tracked YFV-17D replication complexes (presence of both (+) and (−) viral RNA) within immune cell subsets isolated from the blood and spleen of infected WT and stat1^loxP/loxP^/Vav-cre mice (Fig. 5a). Following infection of WT and stat1^loxP/loxP^/Vav-cre mice with YFV-17D, we analysed which murine immune cell subsets are the primary reservoir of YFV-17D replication intermediates in the blood. At day 3 post infection, stat1^loxP/loxP^/Vav-cre mice displayed a significant increase of CD45+ cells carrying YFV-17D replication intermediates, correlating with the levels of viremia at this time point (Fig. 5b). Among all murine CD45+ cells, macrophages (MPH), cytotoxic CD8+ T cells (CTL) and conventional dendritic cells (cDCs) were the main sites of YFV-17D replication when stat1 was knocked-out in the hematopoietic compartment (Fig. 5b and Supplementary Fig. 4a). This burst of YFV-17D replication in hematopoietic cells was transient in nature as YFV-17D replication intermediates could not be detected at day 11 post infection in either WT or stat1^loxP/loxP^/Vav-cre mice (Fig. 5c). An increase in CD8+ T cells carrying viral replication complexes in the periphery was associated with a drop in peripheral CD4+ and CD8+ T cells at day 3 post infection (Fig. 5d), suggesting viral replication in the periphery might affect T cell proliferation and activation. Consistently, mRNA transcripts of the IL-12 receptor and numerous cytokines, including MCP-1, IP-10, IL-6, IFN-β and GM-CSF, were significantly lower in CD8+ T cells enriched from the blood as compared to non-CD8+ cells of stat1^loxP/loxP^/Vav-cre mice at day 3 post infection (Fig. 5e,f and Supplementary Fig. 4b). As CD8+ T cells appeared to be major cytokine producers among peripheral blood mononuclear cells (PBMCs) of stat1^loxP/loxP^/Vav-cre mice (Supplementary Fig. 4c), our results suggest an exacerbated cytokine response could be triggered in a PBMC-independent manner to counteract the defect in the response of peripheral T cells to infection. We then sought to determine whether an increased viral load in the spleen of stat1^loxP/loxP^/Vav-cre mice correlates with an increased level of viral RNA-bearing cells in spleen-resident immune cells. At day 11 post infection, we observed a significant increase in the frequency of cells bearing (+) and (−) strand YFV-17D RNA. Both YFV-17D RNA complexes were significantly increased in spleen-resident CD19+ B cells, CD3− CD161+ NK cells and in the total CD11b+ cell population (that comprises NK cells) of stat1^loxP/loxP^/Vav-cre mice in comparison to WT (Fig. 5g). Among these subsets, only NK cells in which YFV-17D was replicating experienced a significant increase between days 3 and 11 post infection in contrast to the number of YFV-17D replicating-B cells that had a more stable level during this period (Supplementary Fig. 4d). The increase in viral replication observed in the total CD11b+ population was mainly due to NK cells, as the CD11b+ CD161− cell fraction harbouring both (+) and (−) YFV-17D RNA was similar between WT and stat1^loxP/loxP^/Vav-cre mice (data not shown). Taken together, our data suggest a link between an immunopathogenic process and increased YFV-17D replication in tissue-specific immune cell subsets when Stat1 is knocked-out in the murine hematopoietic compartment.

### YFV-17D infects IFN-signalling competent human immune cells

**Figure 6:**
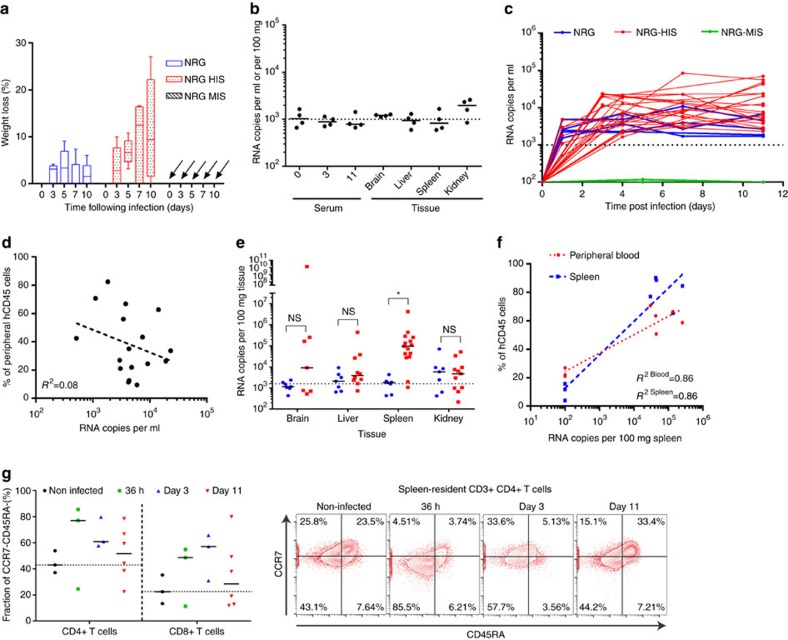
NRG-HIS mice display unique phenotype and replication dynamics following YFV-17D infection. (**a**) Weight loss of non-engrafted NRG mice, NRG-HIS and NRG-MIS mice over the course of YFV-17D infection (Min to Max box and whiskers; NRG and NRG-HIS, *n*=6 per group; NRG-MIS, *n*=4). (**b**) Serum viremia of NRG-MIS mice over the course of infection, and tissue viral loads at killing (day 11 post infection). (+) RNA copies per ml per 100 mg of tissue were quantified by RT-qPCR. Medians and limit of detection (dotted line) are shown (*n*=4). (**c**) Serum viremia of NRG (blue), NRG-HIS (red) and NRG-MIS (green) mice in the peripheral blood over the course of infection. (+) RNA copies per ml were quantified by RT-qPCR. Limit of detection (dotted line) is shown (NRG, *n*=5; NRG-HIS, *n*=24; NRG-MIS=4). (**d**) Quantitative correlation (semilog nonlinear regression, R^2^ is indicated) between the level of peripheral humanization and serum viremia in NRG-HIS mice. Blood of four NRG-HIS mice was collected at multiple times over the course of infection and viremia, as well as peripheral humanization, quantified (*n*=17). (**e**) Viral loads within different tissues of NRG and NRG-HIS mice at day 11 post infection. (+) RNA copies per 100 mg of tissues were quantified by RT-qPCR. Medians and limit of detection (dotted line) are shown (NRG, *n*=7; NRG-HIS, *n*=11–15; *P<0.05, NS non-significant; Two-way ANOVA test). (**f**) Quantitative correlation (semilog nonlinear regression, R^2^ is indicated) between spleen viremia and the level of peripheral and spleen humanization in NRG-HIS mice at day 11 post infection (*n*=8). (**g**) Frequencies of spleen-resident human CD3+CD4+ and CD3+CD8+ T cells negative for CCR7 and CD45RA expression following infection of NRG-HIS mice with YFV-17D. Frequencies are reported for times post infection (36 h, days 3 and 11 post infection). Medians for each time point are shown and a black dotted line represents the median of the non-infected control mice (*n*=3–6). Representative FACS plots of CCR7 and CD45RA expression among spleen-resident human CD3+CD4+ T cells are displayed on the right panel.

YFV has a very restricted host tropism and robust infection in immunocompetent hosts is limited to humans and select non-human primate species. The mechanisms resulting in the robust immunity induced by YFV-17D appear as host-specific as the pathogenesis observed during YFV-Asibi infection. Hence, we aimed to compare YFV-17D replication dynamics in the murine immune system with those in the HIS. To do so, we conducted the first characterization of YFV-17D infection in a humanized mouse model established in our lab^29^,^30^,^31^. Non-obese diabetic (NOD) mice deficient for both recombinase activating gene 1 (Rag1^−/−^) and IL-2 receptor gamma chain (IL2Rγ^null^), also known as NRG mice, were injected with human hematopoietic stem cells (HSCs). These mice reached plateau levels of HIS reconstitution 12 weeks post engraftment, yielding so-called NRG-HIS mice (Supplementary Fig. 5a). YFV-17D was characterized in both NRG-HIS mice and murinized mice, i.e. NRG mice reconstituted with an allogeneic mouse immune system (NRG-MIS), and non-engrafted NRG control mice (Supplementary Fig. 5b). Following YFV-17D infection, NRG-HIS mice exhibited transient weight loss but recovered, maintained a stable body temperature and did not show any other signs of morbidity or any mortality (Fig. 6a and Supplementary Fig. 6a). In contrast, NRG and NRG-MIS mice did not experience weight changes or develop overt signs of clinical disease, but did have considerable temperature fluctuation (Supplementary Fig. 6a). In contrast to stat1^loxP/loxP^/Vav-cre mice, highly immunocompromised NRG mice, which lack functional NK, B, and T cells, did not succumb to YFV-17D infection, demonstrating that cell-intrinsic antiviral defenses are more important for control of the live-attenuated virus *in vivo*. Taken together, our data suggest these observed differences in the course of disease can be attributed to the interaction of YFV-17D with the HIS in a manner distinct from that occurring in murine immune cells. In human YFV-17D vaccines, viral titre increases in the blood over the first days of infection and then decreases over time in a heterogenous manner according to the individual^32^,^33^. Although, no significant serum viremia or tissue viral load was observed in NRG-MIS mice over the course of infection (Fig. 6b), serum viremia in NRG-HIS mice increased rapidly over time following infection and plateaued at low-levels 3 to 4 days post infection, which was distinct from the serum viremia profile of WT and stat1^loxP/loxP^/Vav-cre mice (Fig. 6c). Moreover, viremia in NRG-HIS mice showed heterogeneity similar to that observed in humans^32^. However, in contrast to human vaccinees, NRG-HIS mice do not eventually clear YFV-17D, which is likely a reflection of the limited functionality of the engrafted HIS (refs 29, 34, 35).

In contrast to NRG-MIS mice, which efficiently controlled YFV-17D infection, non-engrafted NRG mice maintained YFV-17D levels for 11 days, but the plateau was 3–20-fold lower than in ∼50% of humanized mice (Fig. 6c). Since only about half of the humanized mice developed higher peripheral viremia than non-engrafted NRG mice, we aimed to determine which experimental parameters within the NRG-HIS cohorts correlated with YFV-17D viremia. Serum viremia in NRG-HIS mice did not correlate with the level of peripheral humanization (Fig. 6d). In addition, there were not significant differences in viral load in the brain, liver and kidney, but YFV-17D RNA was significantly higher in the spleens of infected NRG-HIS as compared to NRG mice (Fig. 6e). These data suggest viral infection of spleen-resident human cells might be an important event during the course of YFV-17D infection. Consistent with this hypothesis, the level of peripheral and spleen humanization correlated with the viral load in the spleen (Fig. 6f). In contrast to stat1^loxP/loxP^/Vav-cre mice, extensive viral replication in the periphery was not associated with high-level secretion of pro-inflammatory cytokines (Supplementary Fig. 6b). Instead, some cytokines involved in adaptive immune response activation (IL-18, IFN-γ) increased over the course of infection.

Consistently, activated CD4+ and CD8+ T cells (CCR7−CD45RA) were observed in the spleen of 8 out of 12 animals over the course of infection (Fig. 6g). Despite heterogeneity in the levels of immune activation across animals, the T cell activation profile over time suggested activation likely occurs early during the course of infection (Fig. 6g). Taken together, our data demonstrate that robust YFV-17D replication is dependent on a primate immune system in the context of functional IFN-dependent, cell-intrinsic immune defenses. Moreover, viral replication in the spleen could be an important event reflecting specific YFV-17D interactions with human immune cells present in the humanized mice.

### Distinct YFV-17D infection dynamics in humanized mice

**Figure 7:**
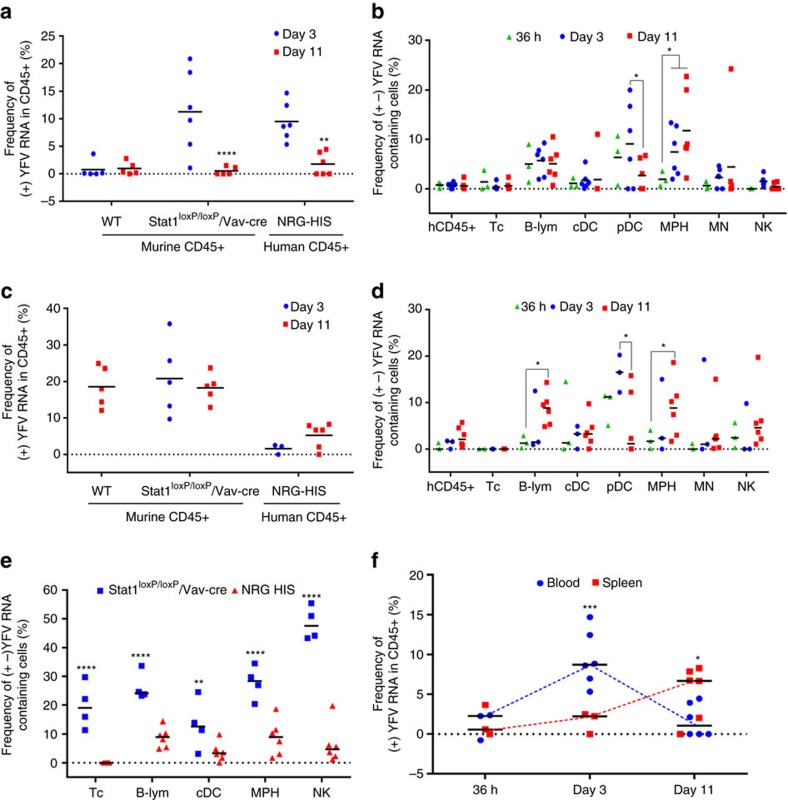
vRNA flow uncovers human-specific viral spatio-temporal dynamics in humanized mice. (**a**) Frequency of peripheral murine and human CD45+ cells containing (+) YFV-17D RNA within the blood of WT, stat1^loxP/loxP^/Vav-cre mice and NRG-HIS mice at day 3 (blue) and 11 (red) post infection. All frequencies were normalized to frequencies determined before infection for each animal (*n*=5–6 per group; **P<0.01, ****P<0.0001; Two-way ANOVA test). (**b**) Frequency of cells containing both (+) and (−) YFV-17D RNA in multiple subsets of peripheral human CD45+ cells isolated from the blood of NRG-HIS mice at 36 h, 3 and 11 days post infection. All frequencies were normalized on frequencies determined before infection for each animal (*n*=3–6 per group; *P<0.05; Two-way ANOVA test). (**c**) Frequency of spleen-resident murine and human CD45+ cells containing (+) YFV-17D RNA strand within WT, stat1^loxP/loxP^/Vav-cre mice and NRG-HIS mice at day 3 (blue) and 11 (red) post infection (*n*=5–6 per group; **P<0.01, ****P<0.0001; Two-way ANOVA test). (**d**) Frequency of cells containing both (+) and (−) YFV RNA in multiple subsets of spleen-resident human CD45+ cells isolated from NRG-HIS mice 36 h, 3 and 11 days post infection (*n*=3–6 per group; *P<0.05). (**e**) Frequency of cells containing both (+) and (−) YFV-17D RNA in five selected spleen-resident immune cell subsets isolated from the spleen of stat1^loxP/loxP^/Vav-cre mice and NRG-HIS mice at day 11 post infection (*n*=3–6 per group; *P<0.05, ****P<0.0001; Two-way ANOVA test). (**f**) Kinetics of the frequency of peripheral and spleen-resident human CD45+ cells containing (+) YFV RNA in NRG-HIS mice over the course of infection (36 h, three and eleven days post infection; *n*=5–6 per group; *P<0.05, ***P<0.001; Two-way ANOVA test). Frequencies of YFV RNA-bearing cells were normalized to background staining in the equivalent cell populations in spleens of non-infected animals. Tc, CD3+ T cells; B-lym, CD19+ B cells; cDC, CD3− CD19− CD11c+ conventional dendritic cells; pDCs, CD3− CD19− CD123+ plasmacytoid dendritic cells; MPH, CD68+ macrophages; MN, CD14+ monocytes; NK, CD3− CD19− CD56+ NK cells. Details in population gating are described in materials and methods.

We then sought to decipher the YFV-17D replication dynamics within the engrafted human immune cells using vRNA flow. After validating the ability of vRNA flow to detect transcripts in human cells isolated from NRG-HIS mice (Supplementary Fig. 6c), we infected cohorts of highly engrafted NRG-HIS mice. These mice subsequently became viremic and maintained significantly higher YFV-17D RNA levels in their sera as compared to non-engrafted mice (Supplementary Fig. 6d). Using vRNA flow, we detected a significant amount of cell-associated (+) viral RNA at day 3 post infection in the blood of NRG-HIS mice, demonstrating that human immune cells fully competent in antiviral signalling pathways are permissive to YFV-17D infection in contrast to WT mice. Of note, the frequencies of (+) viral RNA-bearing cells in the peripheral blood followed kinetics similar to those observed in NRG-HIS mice and stat1^loxP/loxP^/Vav-cre mice (Fig. 7a). This observation is consistent with the idea that mice lacking IFN signalling represent a model that can mimic several features of the YFV pathogenesis and attenuation in humans^27^,^28^. However, in contrast to stat1^loxP/loxP^/Vav-cre mice, in which viral replication complexes were transiently detectable at day 3 (Fig. 5b), (+) and (−) YFV-17D RNA were readily quantifiable in multiple peripheral human cell subsets in NRG-HIS mice at day 11 post infection (Fig. 7b). Viral replication was detectable within 36 h post infection in B cells, plasmacytoid dendritic cells (pDCs) and macrophages (MPHs), and then continued to persist mainly in B cells and MPHs (Fig. 7b and Supplementary Fig. 7a,b). No replication was observed in CD3+ T cells, strengthening the idea of an association between viral replication in CD8+ T cells and pathogenesis in stat1^loxP/loxP^/Vav-cre mice. Overall, these data represent the first piece of evidence that YFV-17D circulates and persists in the periphery both in a cell-free and cell-associated manner in a human context, with a strong affinity for specific PBMC subtypes. Despite the fact that NRG-HIS mice and stat1^loxP/loxP^/Vav-cre mice displayed a similarly elevated viral load in the spleen, the profiles of cell-associated (+) viral RNA over time in the spleen showed major differences (Fig. 7c). At 36 h and 3 days post infection, viral replication intermediates were barely detectable in most spleen-resident cell subsets of NRG-HIS mice, with the exception of pDCs, in contrast to stat1^loxP/loxP^/Vav-cre mice where multiple cell subsets displayed high-levels of viral replication (Fig. 7d and Supplementary Fig. 7c). Viral replication increased on day 11 post infection and was restricted to specific cell subsets, namely B cells and MPHs, similar to observations in the blood of NRG-HIS mice at the same time point (Fig. 7d and Supplementary Fig. 7d). Replication was observed to a lesser extent in NK cells and dendritic cells (DCs). Although replication in spleen-resident B cells, macrophages and, to a lesser extent, DCs and NK cells, seemed to represent a common feature between the mouse and human immune system at day 11 post infection, replication in these cells types was overall lower in human cells (Fig. 7e). No replication was observed in human T cells, and NK cells did not appear to be a major replication reservoir. Cell-associated virus kinetics in human cells displayed a distinct signature characterized by early replication in the peripheral compartment that was then counterbalanced by late, slowly increasing replication in specific spleen-resident cell subsets (Fig. 7f).

## Discussion

Flaviviruses are a cause of major health concern worldwide. The mosquito-mediated transmission of these pathogens, combined with their rapid genetic co-evolution with their host, strongly impacts the incidence of cyclical outbreaks that increase in frequency with climate and environmental changes. However, our lack of knowledge regarding the interactions between these pathogens and their hosts strongly hampers our ability to effectively respond to these outbreaks. The recent outbreaks attributed to ZIKV (refs 36, 37) and YFV (ref. 2), both isolated and identified several decades ago, illustrate such concerns.

**Figure 8:**
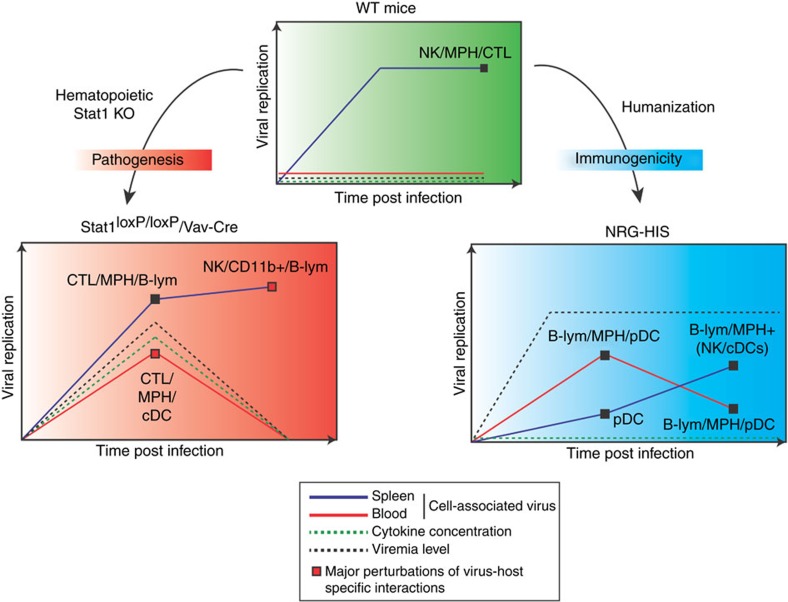
Hallmarks of YFV-17D infection and cellular tropism within the murine and human immune system. Schematic representation of the different dynamics of YFV-17D replication in different immune contexts associated with distinct outcomes of infection (WT, green; stat1^loxP/loxP^/Vav-cre, red; NRG-HIS, blue). Relative amount of cell-associated virus is schematically represented over time within the spleen (dark blue line) and the blood (red line) of each type of animal. For each line and time point, the major cell subset(s) replicating YFV-17D are indicated. Serum viremia level (black dotted line) and serum cytokine concentration (green dotted line) are also represented for each type of animal. CTL, CD8+ T cells; B-lym, CD19+ B cells; human NK, CD3− CD19− CD56+ NK cells; murine NK, CD3− CD161+ NK cells; cDC, CD3− CD19− CD11c+ conventional dendritic cells; human pDCs, CD3− CD19− CD123+ plasmacytoid dendritic cells; human MPH, CD68+ macrophages; murine MPH, CD3− CD19− CD11b+ F4/80+ macrophages. Details in population gating are described in materials and methods.

Today, there is an urgent need to harness novel experimental approaches in order to shed light on the interactions between these pathogens and their hosts. Thus, characterizing interactions between flaviviruses and the immune response, the major defence system against infection, is of great interest. In this study, we applied a flow-cytometric approach to track flavivirus RNA at the single-cell level in different immune systems and functional contexts. We demonstrated how YFV-17D distinctively interacts with cells of the murine and human immune system in different mouse models and how these dynamics are affected by blunted innate immune responses. Moreover, our results provide a broad picture of how the interactions between YFV-17D and the immune system are connected to different outcomes of infection in these experimental models (Fig. 8). We also identified components of the immune system that are key players in these dynamic changes and potentially involved in the mechanisms of pathogenesis or immunogenicity. Furthermore, we identified previously undescribed immune cell subsets permissive to viral replication. However, it should be noted that immune responses in human patients and vaccinees likely differ from those in our experimental mouse models due to the incomplete humanization of the hematopoietic cell compartment in our humanized mice and the general differences between the murine and human immune system^38^.

Following YFV-17D infection, stat1^loxP/loxP^/Vav-cre mice displayed more severe pathogenesis in comparison to WT mice. Viral replication was not observed in immune cell subsets found in the peripheral blood of WT mice, and serum viremia was low or non-detectable. Although viral clearance seemed to occur in the periphery, cell-associated viral replication was observed in several immune cell subsets of the spleen of WT mice at day 11 post infection, suggesting YFV-17D remained constrained by the immune system in the spleen before complete viral clearance. Stat1 knock-out in the hematopoietic compartment rendered multiple peripheral cell-subsets permissive to viral replication early during the course of infection before peripheral clearance, presumably due to the rise in various pro-inflammatory cytokines. In the murine spleen, viral replication was enhanced in B cells and NK cells in the absence of Stat1, and replication in NK cells showed the strongest increase over time. To our knowledge, our findings represent the first evidence of YFV-17D replicating in murine B cells and NK cells. Hence, the increased interactions between YFV-17D and murine immune components and the modified viral replication dynamics triggered by the blunting of innate immunity highlight a potential signature of YFV-17D pathogenesis (Fig. 8 and Supplementary Table 1). Viral replication in the NK and B cells of YFV-infected patients or YFV-17D vaccinees has not been shown and remains to be proven. However, vRNA flow now provides the means to perform such analyses in human patients.

Immunopathology via elevated levels of pro-inflammatory cytokines—known as a cytokine storm—is thought to play a key role in viscerotropic disease, leading to tight junction disruption and increased vascular permeability^39^,^40^. Similar to our observations, elevated levels of specific cytokines such as MCP-1, IL-6 or IP-10 have been observed in patients with fatal YFV infection^23^, in YFV/YFV-17D-infected mice deficient for type I and/or II IFN (refs 27, 28) and in rhesus macaques infected with YFV (ref. 41).

At day 3 post infection, we observed a viral replication burst in peripheral CD8+ T cells, MPHs and DCs that correlated with a peak in peripheral viremia and a strong increase of pro-inflammatory cytokines in the serum. Replication of YFV-17D in human and/or murine DCs and MPHs has been previously reported^14^,^27^,^42^. Employing the highly sensitive vRNA flow methodology, we provide evidence that YFV-17D can also replicate in mouse CD8+ T cells, albeit only in the absence of Stat1. It still needs to be defined whether infectious viral particles are released from infected murine CD8+ T cells, B cells and/or NK cells.

CD8+ T cells are known as major producers of numerous (pro-)inflammatory cytokines during acute viral infections^39^,^43^. Failure of cytotoxic CD8+ T cells to control viral infection can lead to severe immune dysregulation and immunopathology due to overproduction of cytokines like TNF-α or IFN-γ (refs 39, 44). We observed a significant downregulation in the expression of several cytokines and the IL-12 receptor in peripheral CD8+ T cells at day 3 post infection associated with a depression in the frequency of peripheral T cells at the same time point. High-levels of pro-inflammatory cytokines at the same time point could aid in overcoming the defect in T cell response, reducing peripheral viremia to background levels. However, aberrant lymphocyte proliferation in the periphery after day 3 post infection, as well as histopathologic manifestations in the spleen and liver tissues, suggests that this cytokine storm, along with increased viral replication in particular spleen immune cell subsets, might ultimately lead to a detrimental immunopathogenic process in infected stat1^loxP/loxP^/Vav-cre mice. Taken together, our data point towards a unique link between viral replication in CD8+ T cells, the impairment of a CD8+ T cell-mediated immune response and the induction of a pro-inflammatory cytokine storm. Whether fatal cases of YFV infection in humans are also associated with extensive replication in CD8+ T cells remains to be determined.

YFV has a very narrow host tropism^25^. Disease, as well as protective immunity, is restricted to humans and some non-human primate species. Hence, we sought to harness vRNA flow in a human immune context to assess how YFV-17D replication dynamics are associated with immunogenicity. For this purpose, we characterized YFV-17D infection in humanized mice. Certain human immune cells were highly permissive to YFV-17D infection, displaying persistent viremia and cell-associated virus in the periphery. In human PBMCs but not mouse PBMCs, infection occurred even in the context of intact cell-intrinsic antiviral defenses. The amount of (+) viral RNA in the periphery of NRG-HIS mice at day 3 post infection was similar to stat1^loxP/loxP^/Vav-cre mice, suggesting that mice lacking IFN signalling mimic human features of YFV infection, as previously described^27^. Indeed, YFV-17D has been reported to inhibit human Stat1 and Stat2 signalling in infected cells^45^,^46^ but our data suggest that the same viral evasion mechanisms are ineffective in murine cells, as Stat1 sufficient mice were largely resistant to YFV-17D infection. Only, when Stat1 signalling was inhibited, as in stat1^loxP/loxP^/Vav-cre mice, was YFV-17D able to robustly replicate in immune cell subsets.

Despite these similarities, YFV-17D replication in NRG-HIS mice presented two specific features. Cell-associated virus was not observed in the blood of WT and stat1^loxP/loxP^/Vav-cre mice at later time points, but human immune cells bearing replicating viral RNA persisted in the blood of NRG-HIS mice despite a significant drop in replication between days 3 and 11 post infection. In the periphery, viral replication was largely restricted to pDCs, B cells and MPHs, but persisted only in the latter two (Fig. 8 and Supplementary Table 1). Although MPHs and DCs were also major cell types targeted by YFV-17D in the periphery of stat1^loxP/loxP^/Vav-cre mice, no viral replication was observed in peripheral T cells at any time point, underlining that replication in this cell type might be important in pathogenesis.

Second, while the frequency of cells harbouring (+) viral RNA was initially low in the spleens of NRG-HIS mice, it gradually increased over time. Replication was only detected at day 11 post infection and was mostly restricted to cell subsets previously identified in the periphery: B cells, MPHs and pDCs. Replication was also observed to a lesser extent in cDCs and NK cells. In contrast, viral replication in the spleen of WT or stat1^loxP/loxP^/Vav-cre mice did not show a restricted cell tropism, and a high amount of cells bearing (+) viral RNA were detected in these mice at all tested time points. YFV-17D replication thus displayed a specific spatio-temporal signature in NRG-HIS mice, where the blood and the spleen represent, respectively, an early and late replication reservoir containing immune cell subsets similarly targeted within both compartments (Fig. 8 and Supplementary Table 1). Importantly, the higher level of cell-associated virus in the spleens of WT as compared to NRG-HIS mice also suggests host-specific interactions modulate YFV-17D immunogenicity, viral replication and tropism. The perturbation of these interactions, as in the spleen of stat1^loxP/loxP^/Vav-cre mice, might be detrimental for the host and contribute to immunopathogenesis. Hence, investigating how the genetic differences between YFV-17D and YFV virulent strains affect the viral replication signature in the immune system and perturb host-specific interactions could provide important insights into the mechanisms of YFV pathogenesis in humans.

Our *in vivo* data are consistent with previous results demonstrating YFV-17D can replicate in human MPHs, DCs and pDCs^14^,^42^,^47^. This preference for infecting myeloid lineage cells is largely based on observations made in isolated cell populations infected *in vitro*. However, our data go beyond published reports and demonstrate that YFV-17D can replicate in human B cells and—to a lesser extent—NK cells in humanized mice. At this point, it is unknown whether YFV or YFV-17D infects these same cell types in patients/vaccines. In addition, it remains to be proven whether YFV-17D infectious viral particles are produced and released from these cell subsets.

We accumulated multiple lines of evidence suggesting the initiation of an immune response against YFV-17D in NRG-HIS mice. As a major type I IFN producer, pDCs have been shown to be critical in controlling the initial phase of infection^48^. Moreover, YFV-17D's replication in pDCs has been shown to stimulate type I IFN production, hence initiating the immune response^47^. Our results are consistent with these reports as YFV-17D displayed high and early replication in pDCs during the course of infection before a drop in replication at a later time point. Increase of human IFN-γ in humanized mice blood but not in YFV-17D-infected WT and stat1^loxP/loxP^/Vav-cre mice, and the detection of spleen-resident activated T cells are additional markers of an induced immune response in NRG-HIS mice.

Furthermore, replication of YFV-17D in human B cells and MPHs suggests a link between replication in these subsets and the induction of an adaptive immune response in NRG-HIS mice. Indeed, it was recently reported that replication of YFV-17D in human monocyte-derived MPHs stimulate the IFNγ and IL-2 production of CD4+ T cells^14^. Sustained replication in NRG-HIS mice of YFV-17D in human MPHs and the presence of IFNγ in the serum are consistent with this report and strengthen the idea that NRG-HIS mice may develop human-like features of the immune response against YFV-17D. In addition, viral replication in spleen-resident B cells could enhance MHC II-mediated antigen presentation, resulting in the activation and migration of B and helper T cells in the peripheral blood^49^,^50^.

YFV-17D replication in spleen-resident NK cells represents an important difference between NRG-HIS mice and stat1^loxP/loxP^/Vav-cre mice. Indeed, the level of YFV-17D replication in human NK cells remained low over time, but with a dramatic increase in murine NK cells. YFV-17D vaccination in humans induces a strong NK cell response, as evidenced by their activation and proliferation shortly following vaccination^51^. This strong NK cell response was dependent on type I/III IFN signalling^51^. Moreover, increasing evidence indicates an important role for NK cells in mediating memory-like responses and effective vaccine responses^52^. Hence, the presence of type I/III IFN signalling, along with low-levels of viral replication in humans, could be involved in the development of an immunogenicity process (for instance, through Th1 priming and CD8+ T cell response).

Altogether, there is increasing evidence from *ex vivo* studies in patients, *in vitro* experiments and our present study that the human immune system provides a replication reservoir for YFV-17D. We show that infection is controlled in a species-specific manner and promotes the induction of a strong immunogenicity process. In this study, we have demonstrated that tracking viral RNA in a complex cell population is a powerful approach when combined with multiple *in vivo* systems and humanized models. This approach uncovered a broad, yet detailed, overview of flavivirus replication dynamics within multiple immune systems. Moreover, it permitted the association of such dynamics with specific outcomes of infection and identification of mediators and events that potentially regulate the attenuation of YFV-17D.

Although conventional humanized mouse models are unable to develop long-lived, antigen-specific responses or antibody responses^53^, these mice represent a valuable platform for observing the primary adaptive immune response to infection and the mobilization of the different arms of this response. Hence, taking advantage of novel humanized models able to develop strong antigen-specific responses will be of further interest to more deeply characterize the interplays between YFV-17D and the components of the adaptive immune response.

In the future, the tracking of viral RNA in novel humanized mice models, combined with the use of high-throughput sequencing technologies, could further reveal the immune signatures and molecular mechanisms that define viral pathogenicity or attenuation.

## Methods

### Mice

C57BL/6, NOD.Cg-*Rag1*^*tm1Mom*^*IL2rg*^*tmlWjl*^*/SzJ* (ref. 54) (NRG) IL2Rg^null^, B6.Cg-Tg(Vav1-icre)A2Kio/J (ref. 55) (Vav-Cre) and B6.Cg-Tg(Alb-cre)21Mgn/J (Alb-cre (ref. 56)), were obtained from the Jackson Laboratory (Bar Harbor, ME). C57BL/6-Tg(CAG-EGFP)1Osb/J (Jackson laboratory, Bar Harbor, ME) is a kind gift from Yibin Kang (Princeton University, Princeton, NJ). Stat1^loxp/loxp^ mice^57^ were kindly provided by Lothar Hennighausen (National Institute of Health, Bethesda, MD) and backcrossed for 10 generations to the C57BL/6 background. stat1^loxP/loxP^/Vav-cre and stat1^loxP/loxP^/Alb-cre were generated by intercrossing stat1^loxP/loxP^ and Vav-Cre or Alb-Cre mice and typing off-spring with primer combinations distinguishing wild-type and mutant alleles. All animal experiments were performed in accordance to a protocol (number 1930) reviewed and approved by the Institution Animal Care and Use Committee at Princeton University.

### Cells and antibodies

Human HEK293t and Human Huh-7.5 (kindly provided by Charles Rice, Rockefeller University, NY) cells were grown in Dulbecco's modified Eagle's medium (DMEM) supplemented with 10% heat inactivated fetal bovine serum (FBS, Thermo Scientific) and 1% Penicillin Streptomycin (Thermo Scientific). The following anti-mouse Abs were used: From Life Technologies, Invitrogen: CD45-PE-Texas Red clone 30F11 (dilution 1/100); From Biolegends: CD45-PE-Cy7 clone 30-F11 (dilution 1/100), CD3-PECy7 clone 17A2 (dilution 1/100), CD3-PerCP-Cy5.5 clone 17A2 (dilution 1/100), CD4-Alexa Fluor 700 clone V4 (dilution 1/100), Ly6G/Gr1-PerCP-Cy5.5 HK1.4 (dilution 1/50); From BD Biosciences: CD8-V500 clone 53-6.7 (dilution 1/100), CD11c-allophycocyanin clone HL3 (dilution 1/50), CD45RA-PE clone 14.8 (dilution 1/50); From eBiosciences: CD3-Alexa Fluor 700 clone 17A2 (dilution 1/100), CD19-Pe-Cy5.5 clone 1D3 (dilution 1/100), CD161−eFluor450 clone PK136 (dilution 1/50), CD11c-PE-eFluor610 clone N418 (dilution 1/50), CD11b-allophycocyanin-eFluor 780 clone M1/70 (dilution 1/100), F4/80-PE clone BM8 (dilution 1/100), CD317-Alexa Fluor 488 clone eBio927 (dilution 1/50), CD117-PE-Cy7 clone 2B8 (dilution 1/100), TER-119-PerCP-Cy5.5 clone TER-119 (dilution 1/50), CD161−PerCP-Cy5.5 clone PK136 (dilution 1/50), CD197/CCR7-PE clone 4B12 (dilution 1/50), CD27-allophycocyanin-eFluor780 clone LG.7F9 (dilution 1/50), CD28-PerCP-Cy5.5 clone 37.51 (dilution 1/50), CD127-Alexa Fluor 488 clone A7R34 (dilution 1/100), CD62L- allophycocyanin clone DREG-56(dilution 1/50). The following anti-human Abs were used: From BD Biosciences: CD45-V500 clone HI30 (dilution 1/100), CD19-allophycocyanin-Cy7 clone SJ25C1 (dilution 1/100), CD4− allophycocyanin clone RPA-T4 (dilution 1/100), CD8-FITC clone G42-8 (dilution 1/100), CD11c-allophycocyanin clone B-ly6 (dilution 1/50), CD34-FITC clone 581 (dilution 1/100), CD90-PE clone 5E10 (dilution 1/100), CD38-PerCP-Cy5.5 clone HIT2 (dilution 1/100), CD45RA-allophycocyanin clone HI100 (dilution 1/100), HLA-A2-FITC clone BB7.2 (dilution 1/100), HLA-DR-FITC clone G46-6 (dilution 1/100); From Life Technologies, Invitrogen: CD3-PE-Cy5 clone 7D6 (dilution 1/100), CD3-PE-Cy7 clone UCHT1 (dilution 1/100), CD14-Alexa Fluor 700 clone TuK4 (dilution 1/100), CD16-RPE-Texas Red clone 3G8 (dilution 1/100), CD19-Pacific blue clone SJ25-C1 (dilution 1/50); From Biolegends: CD56-allophycocyanin-Cy7 clone HCD56 (dilution 1/100), CD45RA-Alexa Fluor 700 clone HI100 (dilution 1/100); From eBiosciences: CD4-PE clone RPA-T4 (dilution 1/50), CD14-PE-eFluor610 clone 61D3 (dilution 1/50), CD11c-Alexa Fluor 700 clone 3.9 (dilution 1/50), CD123-Pe-Cy5.5 clone 6H6 (dilution 1/50), CD56− eFluor450 clone TULY56 (dilution 1/50), CD68-PE clone Y1/82A (dilution 1/50), HLA-DR-eFluor450 clone L243 (dilution 1/100), CD279/PD-1-allophycocyanin-eFluor780 clone J43 (dilution 1/50), CD38-PE-eFluor610 clone HIT2 (dilution 1/50), CD197/CCR7-PE clone 3D12 (dilution 1/50).

### Isolation of human CD34+ and murine CD117+ HSC

All experiments were performed with authorization from the Institutional Review Board and the IACUC at Princeton University. Human fetal livers (16–22 weeks of gestational age) were procured from Advanced Bioscience Resources (ABR), Inc. (Alameda, CA). Fetal liver was homogenized and incubated in digestion medium (HBSS with 0.1% collagenase IV (Sigma), 40 mM HEPES, 2 M CaCl_2_ and 2 U ml^−1^ DNAse I (Roche) for 30 min at 37 °C. Human CD34+ HSC were isolated using a CD34+ HSC isolation kit (Stem Cell Technologies) according to the manufacturer' protocol. Purification of human CD34+ cells were assessed by quantifying by flow cytometry using an anti-human CD34+-FITC antibody (dilution 1/100, clone 581, BD Biosciences). Expression of human CD90, CD38, CD45RA was assessed among the CD34+ population. Expression of HLA-A2 and HLA-DR1 on total cell population prior and after infection was also quantified. To isolate murine CD117+ cells, femurs and tibia of 7–20 weeks old C57BL/6-Tg(CAG-EGFP)1Osb/J mice (without pre-selection of sex) were flushed with PBS (Life Technologies, Invitrogen). Cells were centrifuged and lysed with 1 × lysis buffer (BD Pharm Lyse, BD Biosciences) for 10 min at room temperature in the dark. Cells were then washed, counted and resuspended at a concentration of 1 × 10^8^ cells per ml in a 2%FBS-1 mM EDTA-PBS solution. CD117+ cells were then isolated using a murine CD117+ HSC isolation kit (Stem Cell Technologies) according to the manufacturer' protocol. Purification of mouse CD117+ cells was assessed by quantifying by flow cytometry before and after the following population: CD45+ GFP+ CD3− CD19− CD161− TER119− Ly6G− CD117+.

### Quantifcation of cytokine expression in murine CD8+ T cells

To isolate murine peripheral CD8+ cells, non-infected and infected (day 3 post infection) stat1^loxP/loxP^ Vav-Cre mice were exsanguinated and total blood were collected. Cells were centrifuged and lysed with 1X lysis buffer (BD Pharm Lyse, BD Biosciences) for 10 min at room temperature in the dark. Cells were then washed, counted and resuspended at a concentration of 1 × 10^8^ cells per ml in a 2%FBS-1 mM EDTA-PBS solution. CD8+ T cells were then isolated using a murine CD8a positive selection kit (Stem Cell Technologies) according to the manufacturer' protocol. Purification of mouse CD8+ T cells were assessed by quantifying by flow cytometry prior and after the following population: CD45+ CD8+. Following enrichment, cells from the CD8+T enriched fraction and from the flow-through were spined and resuspended in RLT lysis buffer (Qiagen). Cellular RNA was then extracted using the RNeasy Mini Kit (Qiagen) following manufacturer's instructions. Cytokine and IL-12R expression were then quantified by one-step RT-qPCR using iTaq Universal SYBR One-step kit (BioRad). Expression was then normalized on the expression of murine HPRT1. Primer sequences are described in Supplementary Table 2.

### Generation of humanized and murinized mice

NRG mice of 1–5 days old were irradiated with 300 cGy and 1.5–2 × 10^5^ human CD34+ or 40,000–50,000 CD117+ HSC were injected intrahepatically 4–6 h after irradiation. Male and female mice transplanted with CD34+ HSC derived from various human donors were used in this study.

### Infectious clone constructs and *in vitro* transcription

pACNR-YFV-17D low-copy number backbone (kindly provided by Charles Rice, Rockefeller University, NY) was transformed and amplified using low recombination NEB 5-alpha high efficiency competent *E. coli* (New England Biolabs). Transformed bacteria were incubated in LB+50 μg ml^−1^ Ampicillin (Sigma-Aldrich) overnight at 30 °C under shaking at 205 r.p.m. Plasmid cDNA was purified using E.Z.N.A. Endonuclease free Maxiprep Kit (Omega), ethanol precipitated and linearized using AFl-II restriction enzyme. Following concentration of linearized DNA by ethanol precipitation, viral RNA was transcribed from 1 μg of linear template using mMESSAGE mMACHINE SP6 kit (Ambion) according to manufacturer's instructions. YFV-17D pol(−) was generated by mutating the RNA-dependent RNA polymerase amino acid motif GDD to a GSA motif (amino acids 3171 to 3173)^58^ using In-Fusion HD cloning kit (Clontech). YFV-17D pol(−) cDNA was then amplified and *in vitro* transcribed as described above. For the production of the YFV-17D [NS4A-3′UTR] positive-sense RNA, the YFV-17D coding sequence from core to NS3 was removed from pACNR-YFV-17D, resulting in pACNR-YFV-17D [NSA4A-3′UTR]. A small [NSA4A-3′UTR] positive-sense viral RNA was then *in vitro* transcribed using this shortened YFV-17D coding cDNA sequence as a template, as described above. For the production of the YFV-17D [NS4A-3′UTR] negative sense RNA, the [NS4A-3′UTR] region of YFV-17D was PCR amplified from the shortened pACNR-YFV-17D [NSA4A-3′UTR]. The obtained PCR fragment was then cloned back in its reverse complement form in the same backbone using in-fusion HD cloning kit (Clontech). The resulting pACNR-YFV-17D [Negative NSA4A-3′UTR] plasmid was then linearized and used as template to produce a small [NSA4A-3'UTR] negative sense viral RNA as described above.

### Transfection of YFV-17D small (+) or (−) sense RNA

HEK293t cells were seeded at a confluency of 3 × 10^5^ in 6 well plates and transfected the day after with either 2 μg of YFV-17D [NSA4A-3′UTR] positive or negative sense viral RNA (*Trans*IT-mRNA Transfection Kit, Mirus). Cells transfected with no viral RNA were used as negative control. Six hours later, cells were processed using viral RNA flow assay and incubated with both YFV-17D (+) and (−) probe sets as described below.

### Electroporation and production of viral stocks

Huh-7.5 cells were washed twice with Opti-MEM Gluta-Max-1 reduced serum media (Life Technologies, Invitrogen) and resuspended at a concentration of 1.5 × 10^7^ cells per ml in Opti-MEM. 2 μg of viral RNA was mixed with 0.4 ml of cell suspension and immediately pulsed in a 2 mm cuvette using a BTX ElectroSquare Porator ECM 830 (860 V, 99 μs, 5 pulses) (BTX). Electroporated cells were incubated at room temperature for 10 min before being dripped into 25 ml (P150 culture dish), 10 ml (P100 dish), 3 ml (6-well plate) or 1 ml (24-well plate) of media. The ratio of one electroporation per P100 dish was maintained, with the quantity of electroporated cells per well/dish scaled to surface area. To produce large scale stock of YFV-17D, 5.4 × 10^7^ Huh7.5 were electroporated with 18 μg of RNA. At 24 h post electroporation, media was changed and replaced by low-serum concentration DMEM (1% FBS). Virus was collected at 48 and 72 h post electroporation. At 72 h post electroporation, virus was polled and concentrated 40 to 100 fold using a Millipore 10,000 MWCO spin filter columns (Merk Millipore) on the last day of collection (3,000 g, 20 min). Viral titre was then assessed using a plaque forming unit assay.

### Titration of viral stocks and YFV-17D *in vitro* infection

To determine the viral titre of the YFV-17D stock, 2.5 × 10^5^ Huh.75 were seeded in a 6-well plate 24 h post infection. Serial dilution from 10^−3^ to 10^−12^ of the viral stock were performed, and 2 ml of each dilution were incubated with Huh7.5 for 6 h at 37 °C. At 6 h post infection, media were replaced by fresh DMEM 10% FBS. Four days post infection, cells were washed with PBS, fixed with 100% ethanol for 25 min and stained with 0.1% crystal violet. The number of plaques for each dilution was then determined. For YFV-17D *in vitro* infection, Huh7.5 were seeded similarly, infected at an MOI of 0.005 and media changed 6 h post infection. Cells were collected at days 1, 2 and 3 post infection for performing YFV RNA flow.

### Mouse infections and monitoring

Three to five month-old C57BL/6, 4 to 8 month-old NOD/Rag1^−/−^/IL2Rg^null^ and 3–9 month-old stat1^loxP/loxP^/Vav or Alb-cre (without pre-selection of sex) were infected through intravenous injection in the tail with 10^6^ pfu of YFV-17D, resuspended in 200 ul of PBS. Clinical manifestations of disease were monitored daily and signs of clinical disease progression recorded through weight, clinical scoring and temperature measurement using a rectal probe. Overall appearance was assessed using a clinical scoring matrix assigned as follows: 0, posture normal, appearance with smooth, shiny fur; 1, posture hunched, appearance with ruffled fur, loss of tone, loss of weight; 2, posture hunched, trembling, shaky, appearance with ruffled fur, loss of weight, rash; 3, posture severely hunched, appearance dishevelled, significant (greater than or equal to 20% weight loss) body weight loss; 4, death. Blood of 200 μl were collected through submandibular bleeding every three days when required by the experimental setting. Serum was separated from blood cells by centrifugation (10 min, 3,500 r.p.m.) for further quantification of serum viremia. For quantification of viremia in humanized mice, all the infected humanized mice displayed a level of engraftment ranging from 25 to 80% out of total CD45+ cells. For the YFV RNA flow experiment, all the infected humanized mice displayed a level of engraftment superior to 40–75%.

### Histologic analysis of liver and spleen tissue

Liver and spleen tissues from non-infected or infected (day 11 post infection) C57BL/6 and stat1^loxP/loxP^/Vav-cre were harvested and incubated in 4% PFA at 4 °C for 48 h. Tissues were then maintained in Ethanol 70%. Tissue processing, paraffin embedding and Hematoxylin and Eosin staining were then performed by HRL histopathology reference laboratory (Hercules, California) under standard procedure. All infected liver and spleen samples from the experimental animals were assessed histologically using hematoxylin-eosin–staining method (formalin fixed). All liver and spleen sections were of sufficient area to permit accurate examination of tissue histopathological manifestations. For each mouse model (WT or stat1^loxP/loxP^/Vav-cre mice), experimental condition (non-infected or infected) and tissue (Liver and Spleen), six tissue sections from three biological replicates (three animals) were examined. Histopathological manifestations observed in infected animal tissues were absent from all examined non-infected animals, and were representative of three biological replicate, for a given type of tissue.

### Isolation of immune cells and organ collection

Mice were bled through submandibular bleeding and 200 μl of blood was collected using EDTA capillary collection tubes (Microvette 600 K3E). Blood cells were then separated from mice serum through centrifugation, and red blood cells were lysed with 1 × lysis buffer (BD Pharm Lyse, BD Biosciences) for 15 min at room temperature in the dark. Following lysis and quenching with 10% FBS DMEM media, blood cells were then washed two times with a 1% FBS-PBS solution before staining. At the time of killing, mice were killed via exsanguination under ketamine/xylazine anaesthesia. For viral RNA quantification, liver, spleen, kidney and brain were collected and placed in individual tubes containing 600 μl of RNAlater solution (Thermo Scientific). Viral RNA extraction procedures are described above. For detection of viral RNA through YFV RNA flow, the spleen was collected and placed in 15 ml of serum-free DMEM. Spleens were then placed in a 6 cm dish, dislocated using a razor blade and digested (0.1% collagenase, Sigma; 40 mM Hepes; 2 mM CaCl_2_; 2 U ml^−1^ DNase1, HBSS, Invitrogen, Life technologies) for 30 min at 37 °C. Following quenching with 10% FBS-DMEM media, splenocytes were strained through a 100 μm strainer and washed with 10% FBS-DMEM two times. Splenocytes were then centrifuged and lysed with 1 × lysis buffer (BD Pharm Lyse, BD Biosciences) for 15 min at room temperature in the dark. Cells were then washed two times with a 1%FBS-PBS solution and counted before staining.

### RNA extraction from serum and tissues

Viral RNA was isolated from mouse serum and Huh-7.5 supernatant using the ZR Viral RNA Kit (Zymo) according to manufacturer's instructions. Total RNA was extracted from Huh7.5 cell pellets and mouse tissues (spleen, brain, liver and kidney) using the RNeasy Mini Kit (Qiagen) following manufacturer's instructions. Isolated tissues (20–30 mg) were suspended in Buffer RLT-1% β-mercaptoethanol (Qiagen), lysed using TissueLyser (Qiagen; 20 cycles per s for 2 min, 1 min wait, 20 cycles per s for 2 min) and centrifuged at high-speed. The resulting supernatant was used for extracting viral RNA.

### YFV-17D single-step RT-quantitative real time PCR

Before quantification, RNA was isolated from cell supernatant and pellets, or from mice serum or tissues as described above. Viral RNA was quantified using single-step RT-quantitative real-time PCR (SuperScript III Platinum One-Step qRT-PCR Kit, Life Technologies, Invitrogen) with primers and Taqman probes targeting a conserved region of the 5′UTR of the 17D genome. Single-step RT*-*qPCR was accomplished in a StepOnePlus Real-Time PCR System (Applied Biosystems) using the following thermal cycling: 52 °C for 15 min, denaturation at 94 °C for 2 min, 40 cycles of denaturation at 94 °C for 15 s, annealing at 55 °C for 20 s and elongation at 68 °C for 20 s. A cDNA sequence coding for the 5′UTR was *in vitro* transcribed and used as standard for the absolute quantification of viral RNA. Primer sequences are described in Supplementary Table 2.

### Antibody staining and flow-cytometry analysis

2–4 × 10^6^ PBMCs, splenocytes or bone marrow derived cells of human or murine origins were isolated as described above and stained for 1 h at 4 °C in the dark with the appropriate antibody cocktail. Following washing (1% FBS in PBS), cells were fixed with fixation buffer (1% FBS, 4% PFA in PBS) for 30 min at 4 °C in the dark. FACS analysis was performed using an LSRII Flow Cytometer (BD Biosciences). Flow cytometry data were analysed using FlowJo software (TreeStar). Chimerism of NRG-MIS mice was assessed by quantifying the following mouse cell population by flow cytometry: CD45+ GFP+ cells, CD45+ GFP+ CD3+ T cells, CD45+ GFP+ CD3+ CD4+ T cells, CD45+ GFP+ CD3+ CD8+ T cells, CD45+ GFP+ CD19+ B cells, CD45+ GFP+ CD161+ NK cells, CD45+ GFP+ CD11c+ dendritic cells, CD45+ GFP+ CD11b+ F4/80+ macrophages. Chimerism of NRG-HIS mice was assessed by quantifying the following human populations: Human CD45+ murine CD45-, CD45+ CD3+ T cells, CD45+ CD3+ CD4+ T cells, CD45+ CD3+ CD4+ CD8+ T cells, CD45+ CD16+ leukocytes, CD45+ CD19+ B cells, CD45+ CD11c+ dendritic cells, CD45+ CD56+ NK/NKT cells, CD45+ CD14+ monocytes. For RNA flow experiments, mouse immune cell subset were gated as followed: T lymphocytes, CD45+ CD3+; CD4+ T cells, CD45+ CD3+ CD4+; CD8+ T cells, CD45+ CD3+ CD8+; B cells, CD45+ CD19+, Natural Killer cells, CD45+ CD3− CD19− CD161+; Conventional dendritic cells, CD45+ CD3− CD19− CD11c+; Plasmacytoid dendritic cells, CD45+ CD3− CD19− CD11c- CD317+; Macrophages, CD45+ CD3− CD19− CD11b+ F4/80+. Human immune cell subsets were gated as followed: T lymphocytes, CD45+ CD3+ CD19-; B cells, CD45+ CD3− CD19+, Natural Killer cells, CD45+ CD3− CD19− CD56+; Conventional dendritic cells, CD45+ CD3− CD19− CD11c+; Plasmacytoid dendritic cells, CD45+ CD3− CD19− CD123+; Monocytes, CD45+ CD14+; Macrophages, CD45+ CD68+. For the characterization of spleen-resident T cell activation, human CD3+ CD4+ and CD8+ T cells were analysed for their expression HLA-DR, CD38, CCR7, CD45RA and PD1. Spleen-resident murine CD3+ CD4+ and CD8+ T cells were analysed for their expression of CD45RA, CCR7, CD27, CD28, CD127 and CD62L. Flow cytometry fluorophor compensation for antibodies was performed using AbC Anti-Mouse Bead Kit (Life Technologies, Invitrogen). Fluorophor compensation for RNA flow labelled probe was performed using UltraComp eBeads and PrimeFlow Compensation Kit (Affymetrix).

### Yellow fever virus prime RNA flow assay

Transfected HEK293t or electroporated Huh7.5 or infected Huh7.5 were trypsinized at different time point post electroporation/infection and washed with PBS. For the characterization of YFV replication dynamics *in vivo*, immune cells from mice blood or spleen were processed and isolated as described above, and cell surface stained with the appropriate antibody cocktail for 1 h at 4 °C. Cells were fixed with a first fixation buffer for 30 min, permeabilized and fixed a second time for 1 h according to manufacturer's instructions (Prime RNA flow, Affymetrix). Following washing, cells were then incubated with both (+) and (−) YFV-17D RNA target probe sets (See Supplementary Table 3 for probe information) for 2 h at 40 °C in a thermocycler combined to a thermolid (Eppendorf) for optimal temperature control, allowing effective target probes hybridization to the respective viral RNA strands. Following several washing steps, cells were successively incubated with Pre-Amplifier and Amplifier molecules. Each incubation was conducted for 1.5 h at 40 °C in a thermomixer (ThermoMixer C, Eppendorf) combined to a thermotop (Eppendorf) for optimal temperature control. Finally, after washing, cells were incubated for 1 h with fluorescent-labelled probe Alexa 647, in the temperature control settings described above. Following several washing steps, antibody and probe fluorescent signals (AL647 for (+) YFV RNA probe set, AL488 for (−) YFV RNA probe set) were then analysed by flow cytometry.

### Cytokine quantification

Cytokine quantification was realized using the LEGENDplex multi-analyte flow assay kit (Biolegend). Serum from C57BL/6, stat1^loxP/loxP^/Vav-cre or NRG-HIS mice was incubated for 2 h at room temperature with customized pre-mixed beads and detection antibodies specific for a panel of 12 murine cytokines (IL-23, IL-12, IFNγ, TNFα, KC, MCP-1, IL1β, IP-10, IL6, IL33, IFNβ and GM-CSF) or for a panel of 13 human cytokines (IL-23, IL-12, IFNγ, TNFα, MCP-1, IL1β, IP-10, IL6, IFNβ, GM-CSF, IFNα2, IL18, IL33). Samples were then incubated for 30 min with SA-PE (Biolegend), wash and resulting fluorescent signals were analysed on a flow cytometer (LSRII, BD Biosciences) according to manufacturer's instructions. Analyte concentration was determined using LEGENDplex software (Biolegend).

### Statistical analysis

GraphPad Prism software (version 6) was used for statistical analysis. A two-way ANOVA with corrected Fisher's Least Significant Difference was used to compare quantitative data (*P<0.05, **P<0.01, ***P<0.001, ****P<0.0001).

### Data availability

All relevant data are available from the authors on request.

## Supplementary Material

Supplementary Information Supplementary Figures and Supplementary Tables

## References

[b1] Butler D. Fears rise over yellow fever's next move. Nature 532, 155–156 (2016). 27075072 10.1038/532155a

[b2] Kupferschmidt K. Infectious diseases. Yellow fever outbreak triggers vaccine alarm. Science 352, 128–129 (2016). 27124428 10.1126/science.352.6282.128

[b3] Haug C. J., Kieny M. P. & Murgue B. The Zika challenge. N. Engl. J. Med. 374, 1801–1803 (2016). 27028782 10.1056/NEJMp1603734

[b4] Sharp P. M. & Hahn B. H. Origins of HIV and the AIDS pandemic. Csh Perspect. Med. 1, a006841 (2011). 10.1101/cshperspect.a006841 PMC3234451 22229120

[b5] Wandeler G., Dufour J. F., Bruggmann P. & Rauch A. Hepatitis C: a changing epidemic. Swiss Med. Wkly 145, w14093 (2015). 25658972 10.4414/smw.2015.14093

[b6] Bhatt S. . The global distribution and burden of dengue. Nature 496, 504–507 (2013). 23563266 10.1038/nature12060 PMC3651993

[b7] Douam F. . Genetic dissection of the host tropism of human-tropic pathogens. Annu. Rev. Genet. 49, 21–45 (2015). 26407032 10.1146/annurev-genet-112414-054823 PMC5075990

[b8] Rall G. F., Lawrence D. M. & Patterson C. E. The application of transgenic and knockout mouse technology for the study of viral pathogenesis. Virology 271, 220–226 (2000). 10860874 10.1006/viro.2000.0337

[b9] Shultz L. D., Brehm M. A., Garcia-Martinez J. V. & Greiner D. L. Humanized mice for immune system investigation: progress, promise and challenges. Nat. Rev. Immunol. 12, 786–798 (2012). 23059428 10.1038/nri3311 PMC3749872

[b10] Gaska J. M. & Ploss A. Study of viral pathogenesis in humanized mice. Curr. Opin. Virol. 11, 14–20 (2015). 25618248 10.1016/j.coviro.2015.01.002 PMC4456257

[b11] Babcock G. J., Decker L. L., Volk M. & Thorley-Lawson D. A. EBV persistence in memory B cells *in vivo*. Immunity 9, 395–404 (1998). 9768759 10.1016/s1074-7613(00)80622-6

[b12] Hazenberg M. D., Hamann D., Schuitemaker H. & Miedema F. T cell depletion in HIV-1 infection: how CD4+ T cells go out of stock. Nat. Immunol. 1, 285–289 (2000). 11017098 10.1038/79724

[b13] Schmid M. A., Diamond M. S. & Harris E. Dendritic cells in dengue virus infection: targets of virus replication and mediators of immunity. Front. Immunol. 5, 647 (2014). 25566258 10.3389/fimmu.2014.00647 PMC4269190

[b14] Cong Y. . Characterization of yellow fever virus infection of human and non-human primate antigen presenting cells and their interaction with CD4(+) T cells. PLoS Neglected Trop. Dis. 10, e0004709 (2016). 10.1371/journal.pntd.0004709 PMC4871483 27191161

[b15] Bowie A. G. & Unterholzner L. Viral evasion and subversion of pattern-recognition receptor signalling. Nat. Rev. Immunol. 8, 911–922 (2008). 18989317 10.1038/nri2436 PMC7097711

[b16] Porichis F. . High-throughput detection of miRNAs and gene-specific mRNA at the single-cell level by flow cytometry. Nat. Commun. 5, 5641 (2014). 25472703 10.1038/ncomms6641 PMC4256720

[b17] Monath T. P. & Vasconcelos P. F. Yellow fever. J. Clin. Virol. 64, 160–173 (2014). 25453327 10.1016/j.jcv.2014.08.030

[b18] Hahn C. S., Dalrymple J. M., Strauss J. H. & Rice C. M. Comparison of the virulent Asibi strain of yellow-fever virus with the 17D vaccine strain derived from it. Proc. Natl Acad. Sci. USA 84, 2019–2023 (1987). 3470774 10.1073/pnas.84.7.2019 PMC304575

[b19] Theiler M. & Smith H. H. The use of yellow fever virus modified by *in vitro* cultivation for human immunization. J. Exp. Med. 65, 787–800 (1937). 19870634 10.1084/jem.65.6.787 PMC2133527

[b20] Bonaldo M. C., Sequeira P. C. & Galler R. The yellow fever 17D virus as a platform for new live attenuated vaccines. Hum. Vaccines Immunotherap. 10, 1256–1265 (2014). 10.4161/hv.28117 PMC4896586 24553128

[b21] Quaresma J. A., Pagliari C., Medeiros D. B., Duarte M. I. & Vasconcelos P. F. Immunity and immune response, pathology and pathologic changes: progress and challenges in the immunopathology of yellow fever. Rev. Med. Virol. 23, 305–318 (2013). 23873723 10.1002/rmv.1752

[b22] Monath T. P. Treatment of yellow fever. Antiviral Res. 78, 116–124 (2008). 18061688 10.1016/j.antiviral.2007.10.009

[b23] ter Meulen J. . Activation of the cytokine network and unfavorable outcome in patients with yellow fever. J. Infect. Dis. 190, 1821–1827 (2004). 15499539 10.1086/425016

[b24] Theiler M. Susceptibility of white mice to the virus of yellow fever. Science 71, 367 (1930). 10.1126/science.71.1840.367 17731835

[b25] Julander J. G. Animal models of yellow fever and their application in clinical research. Curr. Opin. Virol. 18, 64–69 (2016). 27093699 10.1016/j.coviro.2016.03.010

[b26] Jiang X., Dalebout T. J., Lukashevich I. S., Bredenbeek P. J. & Franco D. Molecular and immunological characterization of a DNA-launched yellow fever virus 17D infectious clone. J. Gen. Virol. 96, 804–814 (2015). 25516543 10.1099/jgv.0.000026 PMC4811652

[b27] Meier K. C., Gardner C. L., Khoretonenko M. V., Klimstra W. B. & Ryman K. D. A mouse model for studying viscerotropic disease caused by yellow fever virus infection. PLoS Pathogen 5, e1000614 (2009). 19816561 10.1371/journal.ppat.1000614 PMC2749449

[b28] Thibodeaux B. A. . A small animal peripheral challenge model of yellow fever using interferon-receptor deficient mice and the 17D-204 vaccine strain. Vaccine 30, 3180–3187 (2012). 22425792 10.1016/j.vaccine.2012.03.003 PMC3323739

[b29] Billerbeck E. . Characterization of human antiviral adaptive immune responses during hepatotropic virus infection in HLA-transgenic human immune system mice. J. Immunol. 191, 1753–1764 (2013). 23833235 10.4049/jimmunol.1201518 PMC3735836

[b30] Billerbeck E. . Development of human CD4+FoxP3+ regulatory T cells in human stem cell factor-, granulocyte-macrophage colony-stimulating factor-, and interleukin-3-expressing NOD-SCID IL2Rgamma(null) humanized mice. Blood 117, 3076–3086 (2011). 21252091 10.1182/blood-2010-08-301507 PMC3062310

[b31] Billerbeck E. . Insufficient interleukin-12 signalling favours differentiation of human CD4(+) and CD8(+) T cells into GATA-3(+) and GATA-3(+) T-bet(+) subsets in humanized mice. Immunology 143, 202–218 (2014). 24766459 10.1111/imm.12304 PMC4172137

[b32] Chan K. R. . Cross-reactive antibodies enhance live attenuated virus infection for increased immunogenicity. Nat. Microbiol. 1, 16164 (2016). 10.1038/nmicrobiol.2016.164 PMC7097525 27642668

[b33] Reinhardt B., Jaspert R., Niedrig M., Kostner C. & L'Age-Stehr J. Development of viremia and humoral and cellular parameters of immune activation after vaccination with yellow fever virus strain 17D: a model of human flavivirus infection. J. Med. Virol. 56, 159–167 (1998). 9746073 10.1002/(sici)1096-9071(199810)56:2<159::aid-jmv10>3.0.co;2-b

[b34] Strowig T. . Priming of protective T cell responses against virus-induced tumors in mice with human immune system components. J. Exp. Med. 206, 1423–1434 (2009). 19487422 10.1084/jem.20081720 PMC2715061

[b35] Traggiai E. . Development of a human adaptive immune system in cord blood cell-transplanted mice. Science 304, 104–107 (2004). 15064419 10.1126/science.1093933

[b36] Brown C. Zika virus outbreaks in Asia and South America. Can. Med. Assoc. J. 188, E34 (2016). 26696621 10.1503/cmaj.109-5212 PMC4732977

[b37] Garcia E. . Zika virus infection: global update on epidemiology and potentially associated clinical manifestations. Wkly Epidemiol. Record/Health Sect. Secretariat League Nations 91, 73–81 (2016). 26897760

[b38] Mestas J. & Hughes C. C. Of mice and not men: differences between mouse and human immunology. J. Immunol. 172, 2731–2738 (2004). 14978070 10.4049/jimmunol.172.5.2731

[b39] Tisoncik J. R. . Into the eye of the cytokine storm. Microbiol. Mol. Biol. Rev. 76, 16–32 (2012). 22390970 10.1128/MMBR.05015-11 PMC3294426

[b40] Lee Y. R. . MCP-1, a highly expressed chemokine in dengue haemorrhagic fever/dengue shock syndrome patients, may cause permeability change, possibly through reduced tight junctions of vascular endothelium cells. J. Gen. Virol. 87, 3623–3630 (2006). 17098977 10.1099/vir.0.82093-0

[b41] Engelmann F. . Pathophysiologic and transcriptomic analyses of viscerotropic yellow fever in a rhesus macaque model. PLoS Negl. Trop. Dis. 8, e3295 (2014). 25412185 10.1371/journal.pntd.0003295 PMC4238990

[b42] Barba-Spaeth G., Longman R. S., Albert M. L. & Rice C. M. Live attenuated yellow fever 17D infects human DCs and allows for presentation of endogenous and recombinant T cell epitopes. J. Exp. Med. 202, 1179–1184 (2005). 16260489 10.1084/jem.20051352 PMC2213233

[b43] Jenkins M. R. . Failed CTL/NK cell killing and cytokine hypersecretion are directly linked through prolonged synapse time. J. Exp. Med. 212, 307–317 (2015). 25732304 10.1084/jem.20140964 PMC4354371

[b44] Pennock N. D. . T cell responses: naive to memory and everything in between. Adv. Physiol. Educ. 37, 273–283 (2013). 24292902 10.1152/advan.00066.2013 PMC4089090

[b45] Laurent-Rolle M. . The interferon signaling antagonist function of yellow fever virus NS5 protein is activated by type I interferon. Cell Host Microbe 16, 314–327 (2014). 25211074 10.1016/j.chom.2014.07.015 PMC4176702

[b46] Munoz-Jordan J. L. . Inhibition of alpha/beta interferon signaling by the NS4B protein of flaviviruses. J. Virol. 79, 8004–8013 (2005). 15956546 10.1128/JVI.79.13.8004-8013.2005 PMC1143737

[b47] Bruni D. . Viral entry route determines how human plasmacytoid dendritic cells produce type I interferons. Sci. Signal. 8, ra25 (2015). 25737587 10.1126/scisignal.aaa1552

[b48] Swiecki M. & Colonna M. The multifaceted biology of plasmacytoid dendritic cells. Nat. Rev. Immunol. 15, 471–485 (2015). 26160613 10.1038/nri3865 PMC4808588

[b49] Burstein H. J. & Abbas A. K. T-cell-mediated activation of B cells. Curr. Opin. Immunol. 3, 345–349 (1991). 1716914 10.1016/0952-7915(91)90036-z

[b50] Mooney N. A. . Lymphocyte activation via MHC class II antigens. Nouvelle Revue Francaise D'hematol. 32, 53–57 (1990). 2349082

[b51] Marquardt N. . The human NK cell response to yellow fever virus 17D is primarily governed by NK cell differentiation independently of NK cell education. J. Immunol. 195, 3262–3272 (2015). 26283480 10.4049/jimmunol.1401811

[b52] Rydyznski C. E. & Waggoner S. N. Boosting vaccine efficacy the natural (killer) way. Trends Immunol. 36, 536–546 (2015). 26272882 10.1016/j.it.2015.07.004 PMC4567442

[b53] Theocharides A. P., Rongvaux A., Fritsch K., Flavell R. A. & Manz M. G. Humanized hemato-lymphoid system mice. Haematologica 101, 5–19 (2016). 26721800 10.3324/haematol.2014.115212 PMC4697887

[b54] Brehm M. A. . Parameters for establishing humanized mouse models to study human immunity: analysis of human hematopoietic stem cell engraftment in three immunodeficient strains of mice bearing the IL2rgamma(null) mutation. Clin. Immunol. 135, 84–98 (2010). 20096637 10.1016/j.clim.2009.12.008 PMC2835837

[b55] Ogilvy S. . Promoter elements of *vav* drive transgene expression *in vivo* throughout the hematopoietic compartment. Blood 94, 1855–1863 (1999). 10477714

[b56] Postic C. . Dual roles for glucokinase in glucose homeostasis as determined by liver and pancreatic beta cell-specific gene knock-outs using Cre recombinase. J. Biol. Chem. 274, 305–315 (1999). 9867845 10.1074/jbc.274.1.305

[b57] Klover P. J. . Loss of STAT1 from mouse mammary epithelium results in an increased neu-induced tumor burden. Neoplasia 12, 899–905 (2010). 21076615 10.1593/neo.10716 PMC2978912

[b58] Khromykh A. A., Varnavski A. N., Sedlak P. L. & Westaway E. G. Coupling between replication and packaging of flavivirus RNA: evidence derived from the use of DNA-based full-length cDNA clones of Kunjin virus. J. Virol. 75, 4633–4640 (2001). 11312333 10.1128/JVI.75.10.4633-4640.2001 PMC114216

