# Supplementary material for: Single-cell tracking of flavivirus RNA uncovers species-specific interactions with the immune system dictating disease outcome
Source: Nat Commun. 2017 Mar 14;8:14781. doi: 10.1038/ncomms14781 (PMC5424064; doi:10.1038/ncomms14781)
Supplement: Supplementary Information — Supplementary Figures and Supplementary Tables [file ncomms14781-s1.pdf]

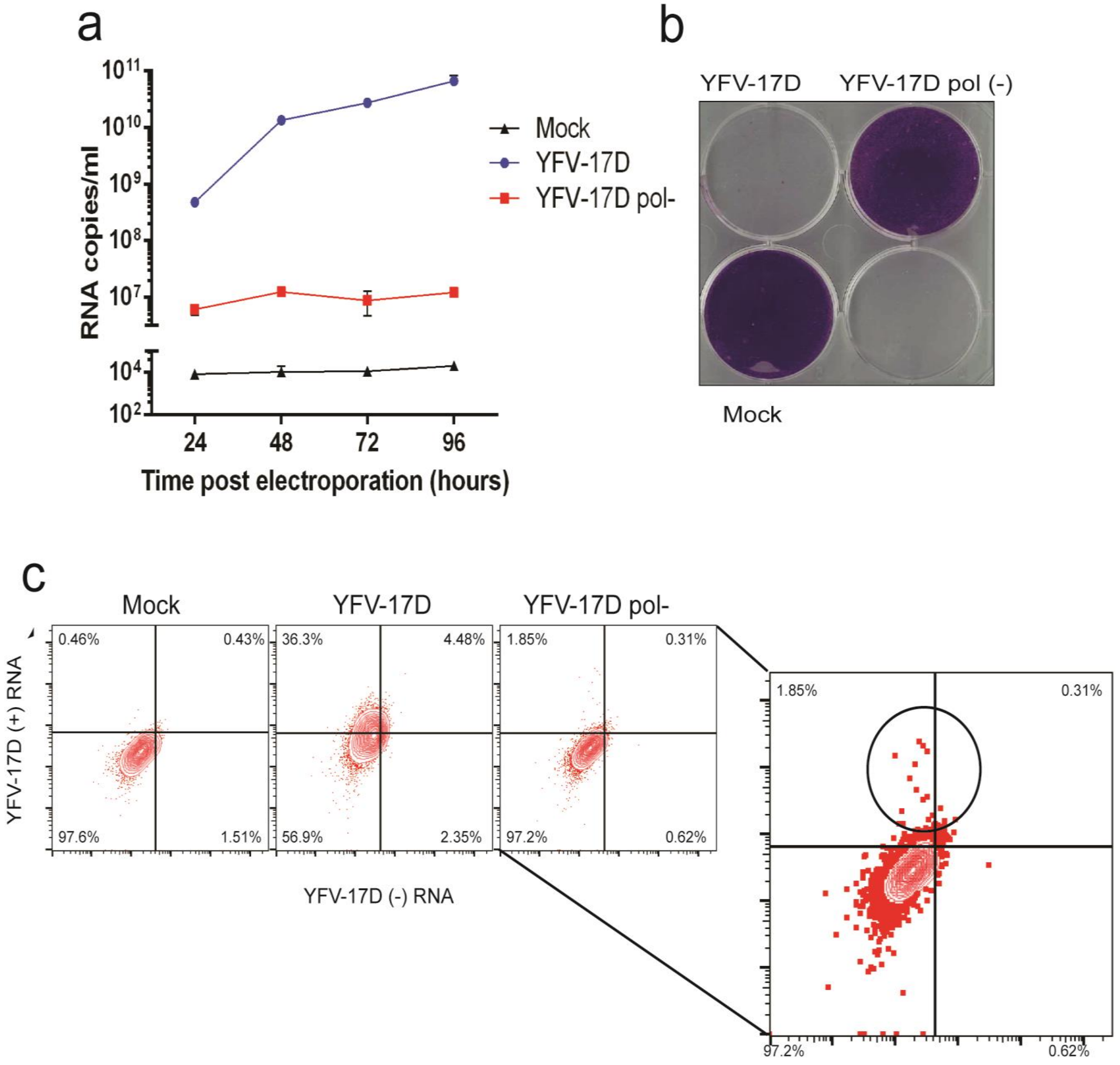

Supplementary Figure 1. a. Quantification by RT-qPCR of YFV-17D and YFV-17D pol- (+) RNA in the supernatant of cultured Huh7.5 cells following viral RNA electroporation of respective viruses over a four days course of infection (mean ± SD; n=3). **b.** Cytopathic effect observed in Huh7.5 four days following electroporation of YFV-17D and YFV-17D pol- RNA. **c.** Representative FACS plots of Huh7.5 electroporated with YFV-17D or YFV-17D pol- RNA and processed using the YFV RNA flow procedure at 36h post electroporation. Cells were stained with both the (+) and (-) YFV RNA probe set. Presence of cells electroporated with YFV-17D pol- and still harboring YFV RNA at 36h post infection is highlighted by a black circle.

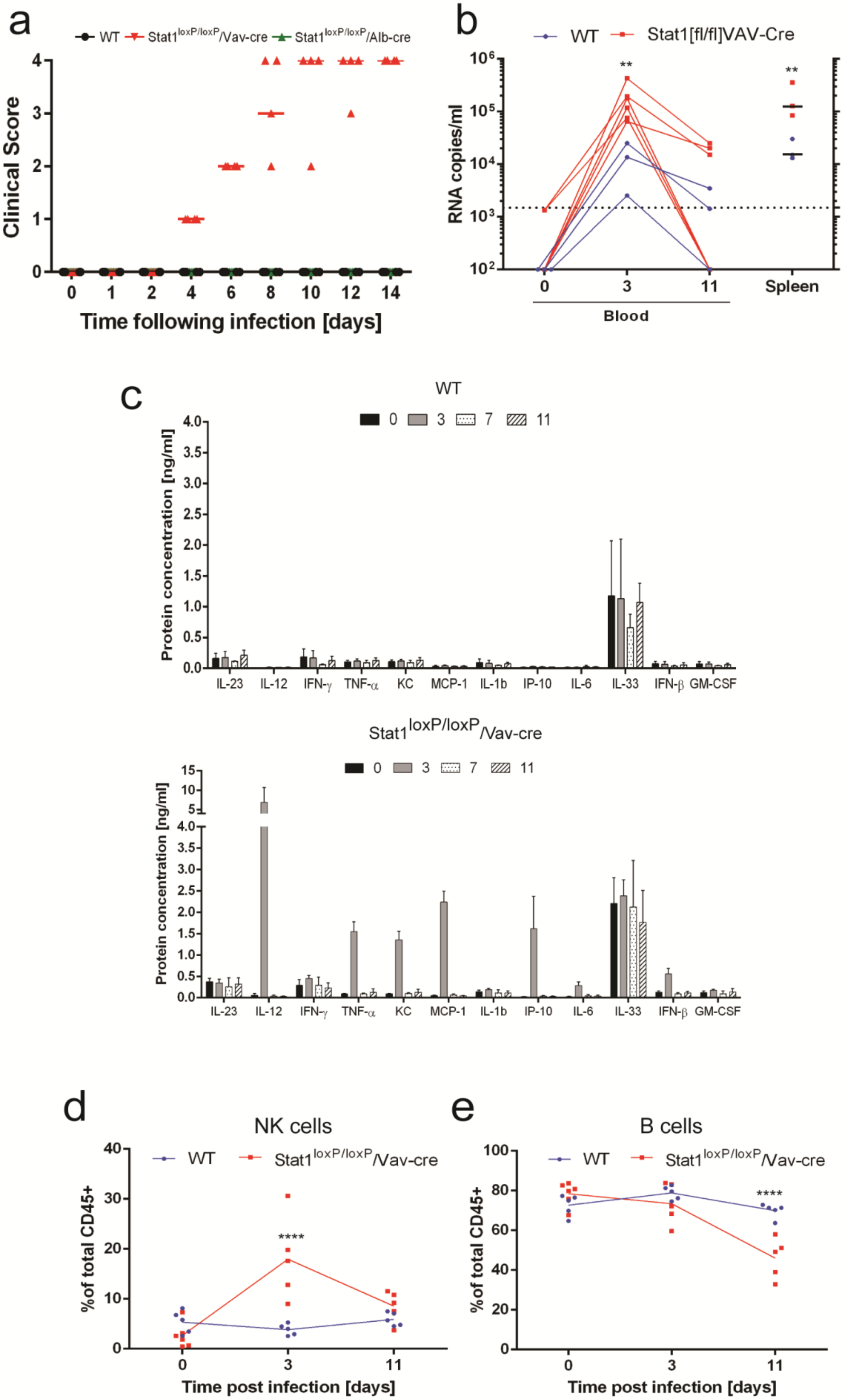

a
WT
Stat1loxP/loxP/Vav-cre
Stat1loxP/loxP/Alb-cre
Clinical Score
0
1
2
3
4
0 1 2 4 6 8 10 12 14
Time following infection [days]
b
WT
Stat1[fl/fl]VAV-Cre
RNA copies/ml
RNA copies/100mg tissue
10^2
10^3
10^4
10^5
10^6
**
**
0
3
11
Spleen
Blood
c
WT
0 3 7 11
Protein concentration [ng/ml]
0.0 0.5 1.0 1.5 2.0 2.5 3.0 3.5 4.0
IL-23 IL-12 IFN-γ TNF-α KC MCP-1 IL-1b IP-10 IL-6 IL-33 IFN-β GM-CSF
Stat1loxP/loxP/Vav-cre
0 3 7 11
Protein concentration [ng/ml]
0.0 0.5 1.0 1.5 2.0 2.5 3.0 3.5 4.0
5
10
15
IL-23 IL-12 IFN-γ TNF-α KC MCP-1 IL-1b IP-10 IL-6 IL-33 IFN-β GM-CSF
d
NK cells
WT
Stat1loxP/loxP/Vav-cre
%of total CD45+
0 10 20 30 40
****
0 3 11
Time post infection [days]
e
B cells
WT
Stat1loxP/loxP/Vav-cre
%of total CD45+
0 20 40 60 80 100
****
0 3 11
Time post infection [days]

Supplementary Figure 2. a. Clinical scoring of C57BL/6 mice (WT), stat1$^{loxP/loxP}$/Alb-cre and stat1$^{loxP/loxP}$/Vav-cre mice over the course of YFV-17D infection (n=5). **b**. Serum viremia over the course of infection (blood) and spleen viral load at day 11 post infection (spleen) of an additional cohort of C57BL/6 mice (WT) and stat1$^{loxP/loxP}$/Vav-cre mice infected with YFV-17D. (+) RNA copies per ml were quantified by RT-qPCR. Medians are shown for the spleen viral loads (n=3-6 per group; ***p*<0.01; Two-way ANOVA test). **c.** Protein concentration of a panel of 12 cytokines in the serum of WT (top) and stat1$^{loxP/loxP}$/Vav-cre (bottom) mice at days 0, 3, 7 and 11 following YFV-17D injection (mean ± SD; n=3). **d-e**. Frequency of peripheral NK cells (**d**) and B cells (**e**) over time following infection of WT (blue) and stat1$^{loxP/loxP}$/Vav-cre mice (red) with YFV-17D. Frequencies are expressed as percentage of total murine CD45+ cells (n=5 per group). Lines are linking the mean of each time point (n=5 per group; *****p*<0.0001;Two-way ANOVA test).

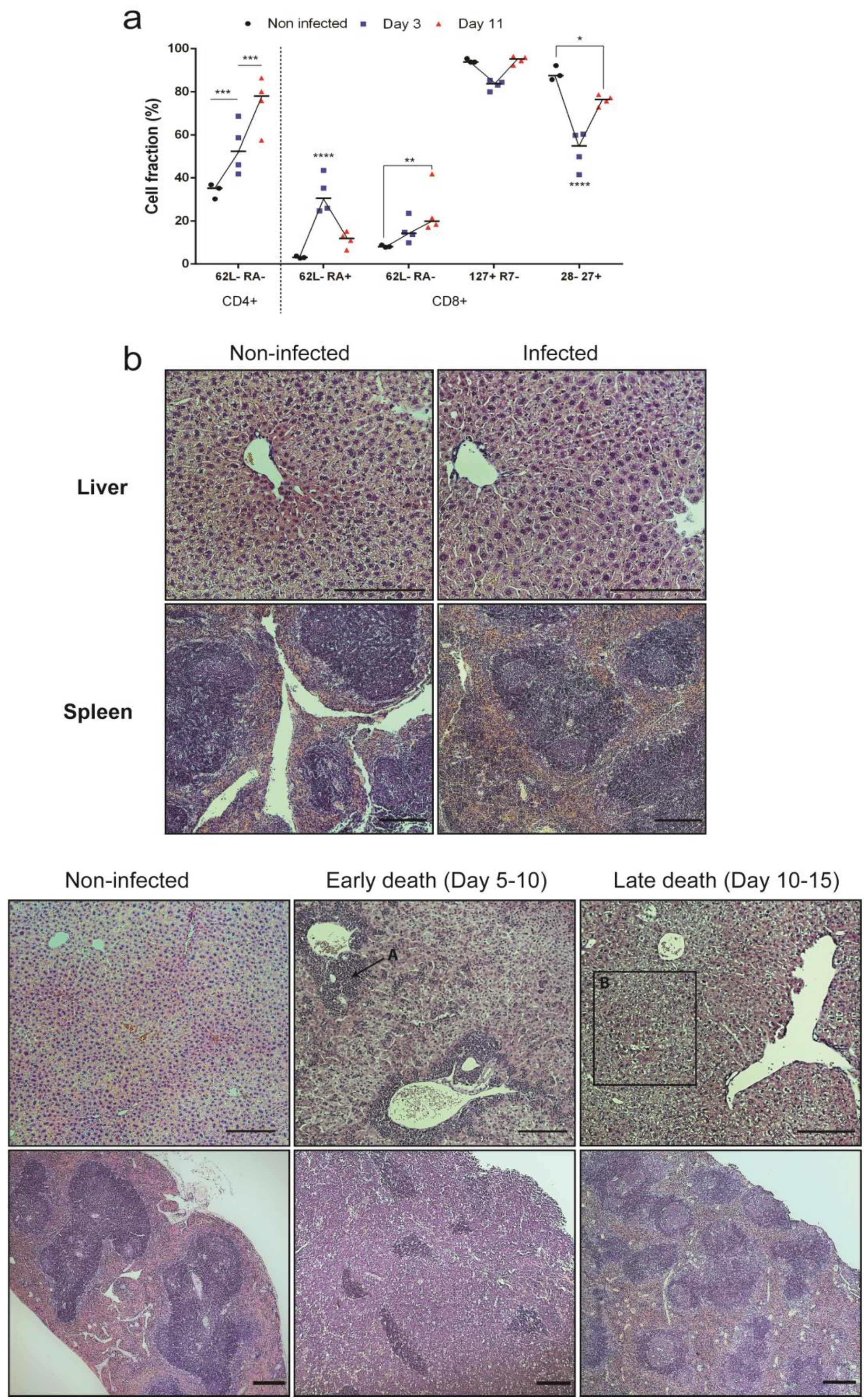
a
Non infected
Day 3
Day 11
Cell fraction (%)
100
80
60
40
20
0
***
***
****
**
*
****
62L- RA-
62L- RA+
62L- RA-
127+ R7-
28- 27+
CD4+
CD8+
b
Non-infected
Infected
Liver
Spleen
c
Non-infected
Early death (Day 5-10)
Late death (Day 10-15)
A
B
Liver
Spleen

Supplementary Figure 3. a. Evolution of expression of CD62L, CD45RA, CD127, CCR7, CD27 and CD28 surface markers on spleen-resident CD3+CD4+ and CD3+CD8+ T cells isolated from non-infected or YFV-17D infected stat1$^{loxP/loxP}$/Vav-cre mice over the course of infection (days 3 and 11 post infection). Medians are shown and linked by a black line for each marker combination (n=3-4 per group; **p*<0.5, ***p*<0.01, ****p*<0.001, *****p*<0.0001; Two-way ANOVA test). **b.** Hematoxylin and eosin staining of liver (20x magnification) and spleen (10x magnification) tissue sections from non-infected and YFV-17D infected WT mice (day 11 post infection). **c.** Hematoxylin and eosin staining of liver (10x magnification) and spleen (4X magnification) tissue sections from non-infected and YFV-17D infected stat1$^{loxP/loxP}$/Vav-cre mice experiencing early (day 3-5 post infection) or late death (day 10-15 post infection). For each experimental condition (non-infected or infected) and tissue (Liver and Spleen), 6 tissue sections from three biological replicates (3 animals) were examined. Histopathological manifestations observed in infected animal tissues were abscent from all examined non-infected animals, and were representative of three biological replicate, for a given type of tissue. Scale bar (200µm) are indicated for each picture. A, severe portal inflammation; B, representative area displaying hydropic changes.

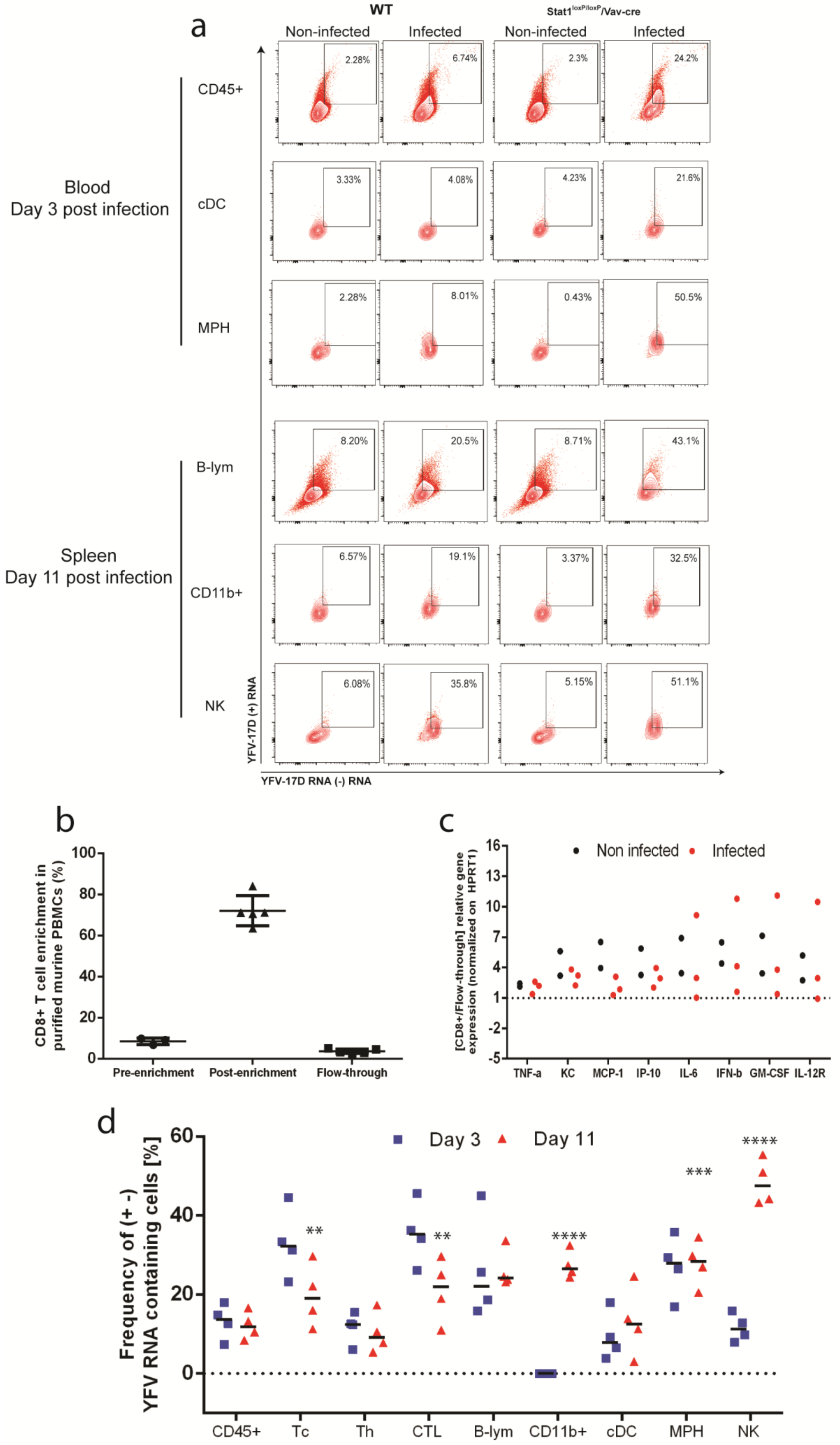

a
WT
Stat1loxP/loxP/Vav-cre
Non-infected
Infected
Non-infected
Infected
Blood
Day 3 post infection
CD45+
cDC
MPH
Spleen
Day 11 post infection
B-lym
CD11b+
NK
YFV-17D (+) RNA
YFV-17D RNA (-) RNA
b
CD8+ T cell enrichment in purified murine PBMCs (%)
Pre-enrichment
Post-enrichment
Flow-through
c
[CD8+/Flow-through] relative gene expression (normalized on HPRT1)
Non infected
Infected
TNF-a
KC
MCP-1
IP-10
IL-6
IFN-b
GM-CSF
IL-12R
d
Frequency of (+ -) YFV RNA containing cells [%]
Day 3
Day 11
CD45+
Tc
Th
CTL
B-lym
CD11b+
cDC
MPH
NK

Supplementary Figure 4. a. Representative FACS plots displaying the frequencies of multiple peripheral (blood, top panel) or spleen-resident (spleen, bottom panel) immune cell subsets containing both (+) and (-) YFV-17D RNA following YFV-17D infection of WT and stat1$^{loxP/loxP}$/Vav-cre mice. Frequencies from non-infected mice are also displayed. **b**. Enrichment efficiency of CD3+ CD8+ T cells from total blood of stat1$^{loxP/loxP}$/Vav-cre mice in a non-infected or infected setting. Frequencies before (pre-enrichment), after (post-enrichment) and of the flow-through are displayed. (mean ± SD; n=5). **c**. Enrichment in CD3+ CD8+ T cells of mRNA coding for multiple cytokines and cytokine receptors. CD3+ CD8+ were isolated from non-infected and YFV-17D infected stat1$^{loxP/loxP}$/Vav-cre and the expression ratio for multiple cytokines and cytokine receptors between the CD8+ enriched fraction and flow-through was determined. Expression of each gene was normalized to HRPT1 expression prior to ratio calculation. (mean ± SD; n=5). **d**. Frequency of cells containing both (+) and (-) YFV RNA in multiple subsets of spleen-resident murine CD45+cells of stat1$^{loxP/loxP}$/Vav-cre mice (red) at days 3 and 11 post infection. (n=5-6 per group; ***p*<0.01, ****p*<0.001, *****p*<0.0001; Two-way ANOVA test). Tc, CD3+ T cells; Th, CD4+ T cells; CTL, CD8+ T cells; B-lym, CD19+ B cells; CD11b+, CD3- CD19- CD11b+ cells; cDC, CD3- CD19- CD11c+ conventional dendritic cells; MPH, CD3- CD19- CD11b+ F4/80+ macrophages; NK, CD3- CD19- CD161+ NK cells. Details in population gating are described in materials and methods.

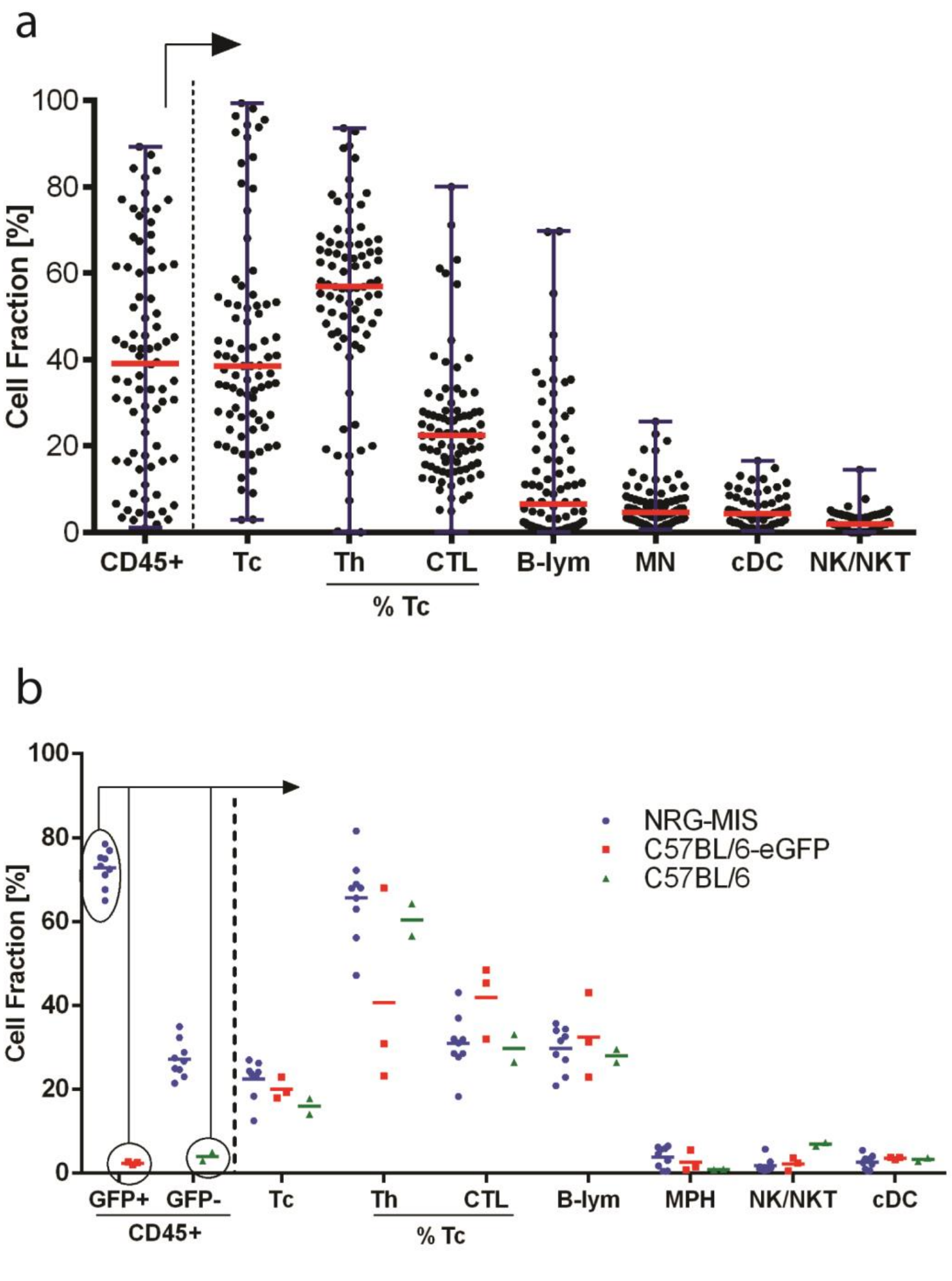

Supplementary Figure 5. Immune system reconstitution in NRG-HIS (**a**, n=82.) and NRG-MIS (**b,** n=9) mice. Frequency of each cell fraction is shown as percentage of CD45+ cells, with the exception of Th and CTL, which are displayed as a percentage of CD3+ T cells. Medians are shown for each cell subset frequency. In panel b, the immune cell subset frequencies of NRG-MIS (blue), C57BL/6-eGFP (red) and C57BL/6 (green) are shown. For each mouse strain, the CD45+ cell population analyzed is circled (CD45+ GFP+ cells or CD45+ GFP- cells). T-lym, CD3+ T cells; Th, CD3+ CD4+ T cells; CTL, CD3+ CD8+ T cells; cDC, CD11c+ conventional dendritic cells; B-lym, CD19+ B cells; Human MN, human CD14+ monocytes; Murine MPH, murine CD11b+ F4/80+ macrophages; Human NK/NKT, human CD56+ NK/NKT cells; Murine NK, murine CD161+ NK cells. Details in population gating are described in materials and methods.

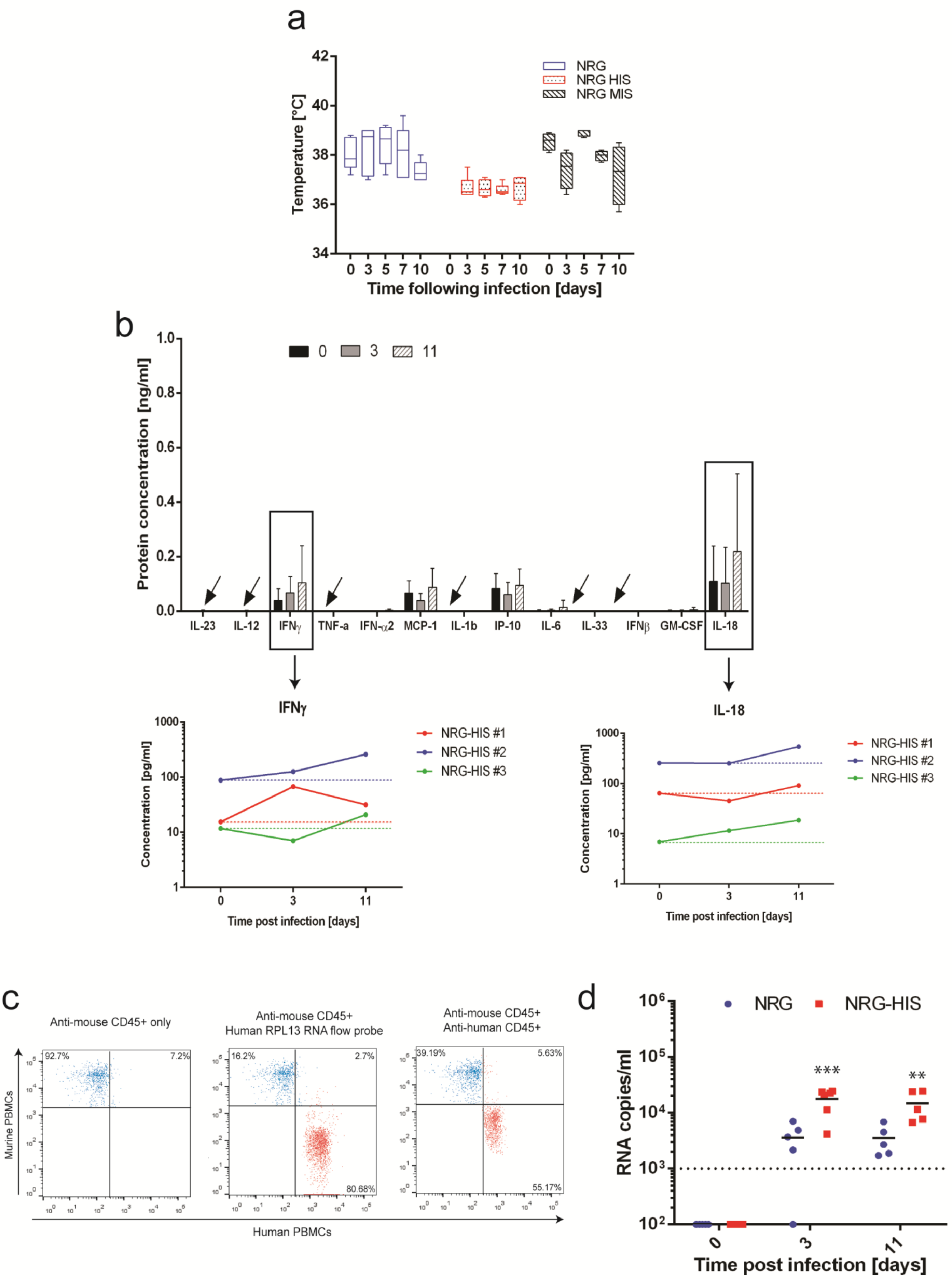
a
Temperature [°C]
NRG
NRG HIS
NRG MIS
Time following infection [days]
b
Protein concentration [ng/ml]
0
3
11
IL-23
IL-12
IFNγ
TNF-a
IFN-α2
MCP-1
IL-1b
IP-10
IL-6
IL-33
IFNβ
GM-CSF
IL-18
IFNγ
Concentration [pg/ml]
NRG-HIS #1
NRG-HIS #2
NRG-HIS #3
Time post infection [days]
IL-18
Concentration [pg/ml]
NRG-HIS #1
NRG-HIS #2
NRG-HIS #3
Time post infection [days]
c
Anti-mouse CD45+ only
Anti-mouse CD45+
Human RPL13 RNA flow probe
Anti-mouse CD45+
Anti-human CD45+
92.7%
7.2%
16.2%
2.7%
80.68%
39.19%
5.63%
55.17%
Murine PBMCs
Human PBMCs
d
NRG
NRG-HIS
RNA copies/ml
***
**
Time post infection [days]

Supplementary Figure 6. a. Body temperature variation in NRG, NRG-HIS and NRG-MIS mice over the course of YFV-17D infection (Min to Max box and whiskers NRG and NRG-HIS, n=6 per group; NRG-MIS, n=4). **b**. Protein concentration of a panel of 13 human cytokines in the serum of NRG-HIS mice at days 0, 3 and 11 following YFV-17D infection. Cytokine concentrations of IFNγ and IL-18 in the serum of each NRG-HIS mice (NRG-HIS #1-3) over the course of infection are also shown. Dotted lines represent the cytokine concentration prior to infection for each animal. (mean ± SD; n=3). **c**. Total human and murine PBMCs of NRG-HIS mice were stained with anti-mouse CD45+ only (left), with anti-mouse CD45+ in combination with anti-human CD45+ (middle) or in combination with a human probe targeting RPL13 mRNA (right). Data are representative of three experiments. **d**. Serum viremia of NRG-HIS mice (red) used for YFV RNA flow at days 3 and 11 post infection. Serum viremia is compared to one of the non-engrafted NRG mice (blue) at similar time points. (+) RNA copies per ml of tissues were quantified by RT-qPCR. Medians are shown (n=5-6 per group; ***p*<0.01, ****p*<0.001; Two-way ANOVA test).

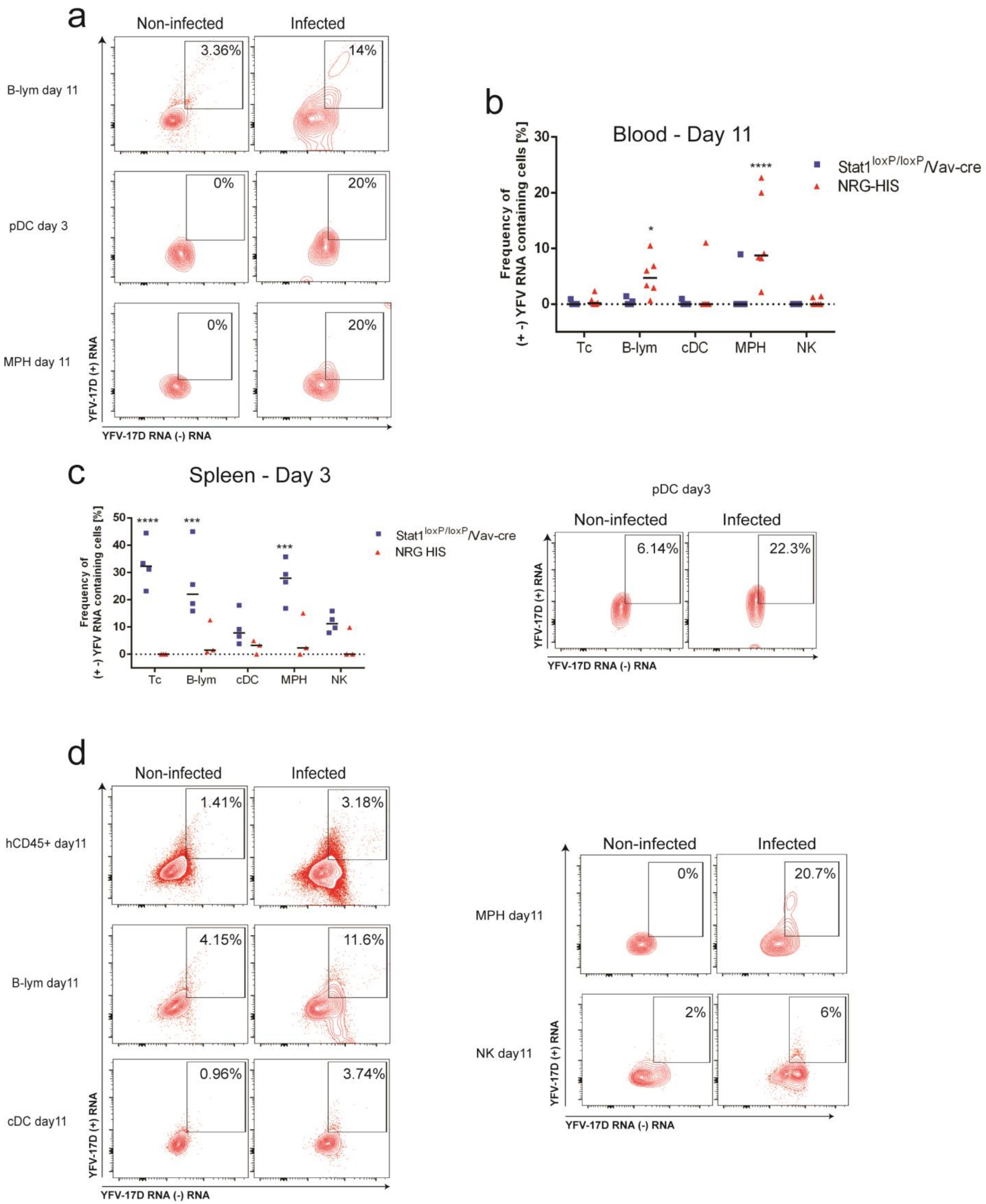

a
Non-infected
Infected
B-lym day 11
3.36%
14%
pDC day 3
0%
20%
MPH day 11
0%
20%
YFV-17D (+) RNA
YFV-17D RNA (-) RNA
b
Blood - Day 11
Frequency of (+ -) YFV RNA containing cells [%]
Stat1 loxP/loxP/Vav-cre
NRG-HIS
Tc
B-lym
cDC
MPH
NK
c
Spleen - Day 3
Frequency of (+ -) YFV RNA containing cells [%]
Stat1 loxP/loxP/Vav-cre
NRG HIS
Tc
B-lym
cDC
MPH
NK
pDC day3
Non-infected
Infected
6.14%
22.3%
YFV-17D (+) RNA
YFV-17D RNA (-) RNA
d
Non-infected
Infected
hCD45+ day11
1.41%
3.18%
B-lym day11
4.15%
11.6%
cDC day11
0.96%
3.74%
YFV-17D (+) RNA
YFV-17D RNA (-) RNA
Non-infected
Infected
MPH day11
0%
20.7%
NK day11
2%
6%
YFV-17D (+) RNA
YFV-17D RNA (-) RNA

Supplementary Figure 7. a. Representative FACS plots displaying the frequencies of selected peripheral immune cell subsets containing both (+) and (-) YFV-17D RNA following YFV-17D infection of NRG-HIS mice. Frequencies from non-infected mice are also displayed. **b.** Frequency of cells containing both (+) and (-) YFV-17D RNA in five selected peripheral immune cell subsets isolated from the blood of stat1$^{loxP/loxP}$/Vav-cre mice and NRG-HIS mice at day 11 post infection. All frequencies were normalized to frequencies determined prior to infection for each animal (n=3-6 per group; **p*<0.05, *****p*<0.0001; Two-way ANOVA test). **c.** Frequency of cells containing both (+) and (-) YFV-17D RNA from five selected spleen-resident immune cell subsets isolated from the spleen of stat1$^{loxP/loxP}$/Vav-cre mice and NRG-HIS mice at day 3 post infection. Frequencies of YFV RNA-bearing cells were normalized to background staining in the equivalent cell populations in spleens of non-infected animals. Representative FACS plots displaying the frequencies of spleen-resident pDCs containing both (+) and (-) YFV-17D RNA following YFV-17D infection of NRG-HIS mice are shown at the right of the panel. Frequencies from non-infected mice are also displayed. (n=3-6 per group; ****p*<0.001, *****p*<0.0001; Two-way ANOVA test). **d.** Representative FACS plots displaying the frequencies of selected spleen-resident immune cell subsets containing both (+) and (-) YFV-17D RNA at day 11 post infection following YFV-17D infection of NRG-HIS mice. Frequencies from non-infected mice are also displayed. Tc, CD3+ T cells; B-lym, CD19+ B cells; cDC, CD3- CD19- CD11c+ conventional dendritic cells; pDCs, CD3- CD19- CD123+ plasmacytoid dendritic cells; MPH, CD68+ macrophages; NK, CD3- CD19- CD56+ NK cells.

		WT	$stat1^{loxP/loxP}$/Vav-cre	NRG-HIS
**Serologic parameters**	Viremia	Low/N.D.	Severe viremia at day 3, followed by a drop toward viral clearance	Persistence over time
	Cytokine	Low/N.D.	Peak at day 3 of pro-inflammatory cytokine	IL-18, IFNγ
**Cell tropism**	General profile	Large tropism	Large tropism	Restricted tropism
	T cells	Spleen only	Increased permissiveness in periphery	N.D.
	NK cells	Spleen mainly	Increased permissiveness in spleen	Spleen, minor
	B cells	Spleen only	Increased permissiveness in spleen	Spleen and periphery
	MPH	Spleen and periphery	Increased permissiveness in periphery	Spleen and periphery
	cDC	Spleen, minor	Increased permissiveness in periphery	Spleen, minor
	pDC	-	-	Spleen and periphery
**Evolution and persistence of cell associated virus in periphery**	In periphery	Very low/N.D.	Peak and clearance	Peak at day 3 and persistence
	In spleen	Maintenance	Maintenance with increased replication in NK cells and B cells	Slow increase over time
**Immunological parameters**	Evolution of T cell frequency in periphery	Stable	Drop and strong increase	Stable
	Presence of activated T cells in spleen	Yes	Yes; More Tem T cells	Yes

Supplementary table 1. Cellular, immunological and serological features characterizing YFV-17D infection in WT mice, $stat1^{loxP/loxP}$/Vav-cre mice and NRG-HIS mice. In the cell tropism section, compartments (Periphery and/or Spleen) where each cell subsets is found to be permissive for YFV-17D replication is indicated. When the cell subset is a minor target in a specific compartment, the compartment is followed by "minor". MPH, macrophages; cDC, conventional dendritic cells; pDC, plasmacytoid dendritic cells; Tem, effector memory T cells. N.D., non-detected; -, undetermined.

**YFV-17D sense 1**	GCTAATTGAGGTGCATTGGTCTGC
**YFV-17D sense 2**	GCTAATTGAGGTGTATTGGTCTGC
**YFV-17D antisense 1**	CTGCTAATCGCTCAACGAACG
**YFV-17D antisense 2**	CTGCTAATCGCTCAAAGAACG
**YFV-17D probe**	ATCGAGTTGCTAGGCAATAAACAC
**Mouse HPRT1 sense**	CCTGGCGTCGTGATTAGTGAT
**Mouse HPRT1 antisense**	AGACGTTCAGTCCTGTCCATAA
**Mouse IL-12R sense**	ATGGCTGCTGCGTTGAGAA
**Mouse IL-12R antisense**	AGCACTCATAGTCTGTCTTGGA
**Mouse TNF-α sense**	CCCTCACACTCAGATCATCTTCT
**Mouse TNF-α antisense**	GCTACGACGTGGGCTACAG
**Mouse KC sense**	CTGGGATTCACCTCAAGAACATC
**Mouse KC antisense**	CAGGGTCAAGGCAAGCCTC
**Mouse MCP-1 sense**	TTAAAAACCTGGATCGGAACCAA
**Mouse MCP-1 antisense**	GCATTAGCTTCAGATTTACGGGT
**Mouse IP-10 sense**	CCAAGTGCTGCCGTCATTTTC
**Mouse IP-10 antisense**	GGCTCGCAGGGATGATTTCAA
**Mouse IL-6 sense**	TAGTCCTTCCTACCCCAATTTCC
**Mouse IL-6 antisense**	TTGGTCCTTAGCCACTCCTTC
**Mouse IFNβ sense**	CAGCTCCAAGAAAGGACGAAC
**Mouse IFNβ antisense**	GGCAGTGTAACTCTTCTGCAT
**Mouse IL-33 sense**	TCCAACTCCAAGATTTCCCCG
**Mouse IL-33 antisense**	CATGCAGTAGACATGGCAGAA
**Mouse GM-CSF sense**	GGCCTTGGAAGCATGTAGAGG
**Mouse GM-CSF antisense**	GGAGAACTCGTTAGAGACGACTT
**Vav-cre genotyping sense**	TCCTGGGCATTGCCTACAAC
**Vav-cre genotyping antisense**	CTTCACTCTGATTCTGGCAATTTCG
**Vav-cre genotyping probe**	ACCCTGCTGCGCATTG
**Alb-cre genotyping sense**	GCGGTCTGGCAGTAAAAACTATC
**Alb-cre genotyping antisense**	GTGAAACAGCATTGCTGTCACTT
**Albv-cre genotyping probe**	AAACATGCTTCATCGTCGGTCCGG

Supplementary table 2. List of primers used in this study.

Virus	RNA strand targeted	nucleotide sequence targeted	Affymetrix Probe I.D.
YFV-17D	Positive strand	8144-9896 (1753bp)	VF1-17153
YFV-17D	Negative strand	9910-10838 (929bp) / 7234-8073 (840bp)	VF4-20655

Supplementary table 3. Information related to the vRNA flow probes used in this study.
